# Structural Requirements of Alkylglyceryl-l-Ascorbic Acid Derivatives for Melanogenesis Inhibitory Activity

**DOI:** 10.3390/ijms19041144

**Published:** 2018-04-10

**Authors:** Norihisa Taira, Yushi Katsuyama, Masato Yoshioka, Osamu Muraoka, Toshio Morikawa

**Affiliations:** 1 SEIWA KASEI CO, LTD., 1-2-14, Nunoichicho, Higashi-osaka, Osaka 579-8004, Japan; katsuyama_yushi@seiwakasei.co.jp (Y.K.); yoshioka_masato@seiwakasei.co.jp (M.Y.); 2 Pharmaceutical Research and Technology Institute, Kindai University, 3-4-1 Kowakae, Higashi-osaka, Osaka 577-8502, Japan; muraoka@phar.kindai.ac.jp (O.M.); morikawa@kindai.ac.jp (T.M.); 3 Antiaging Center, Kindai University, 3-4-1 Kowakae, Higashi-osaka, Osaka 577-8502, Japan

**Keywords:** alkylglyceryl-l-ascorbic acid, melanogenesis inhibitor, 3-*O*-(2,3-dihydroxypropyl)-2-*O*-hexyl-l-ascorbic acid, 2-*O*-(2,3-dihydroxypropyl)-3-*O*-hexyl-l-ascorbic acid, structural requirement, mechanism of action

## Abstract

l-Ascorbic acid has multifunctional benefits on skin aesthetics, including inhibition of melanin production, and is widely used in cosmetics. It, however, has low stability and poor skin penetration. We hypothesize that alkylglyceryl-l-ascorbic acid derivatives, highly stable vitamin C–alkylglycerol conjugates, would have similar anti-melanogenic activity with better stability and penetration. We test 28 alkylglyceryl-l-ascorbic acid derivatives (**1**–**28**) on theophylline-stimulated B16 melanoma 4A5 cells to determine if they inhibit melanogenesis and establish any structure–function relationships. Although not the most potent inhibitors, 3-*O*-(2,3-dihydroxypropyl)-2-*O*-hexyl-l-ascorbic acid (**6**, IC_50_ = 81.4 µM) and 2-*O*-(2,3-dihydroxypropyl)-3-*O*-hexyl-l-ascorbic acid (**20**, IC_50_ = 117 µM) are deemed the best candidate derivatives based on their inhibitory activities and low toxicities. These derivatives are also found to be more stable than l-ascorbic acid and to have favorable characteristics for skin penetration. The following structural requirements for inhibitory activity of alkylglyceryl-l-ascorbic acid derivatives are also determined: (i) alkylation of glyceryl-l-ascorbic acid is essential for inhibitory activity; (ii) the 3-*O*-alkyl-derivatives (**2**–**14**) exhibit stronger inhibitory activity than the corresponding 2-*O*-alkyl-derivatives (**16**–**28**); and (iii) derivatives with longer alkyl chains have stronger inhibitory activities. Mechanistically, our studies suggest that l-ascorbic acid derivatives exert their effects by suppressing the mRNA expression of tyrosinase and tyrosine-related protein-1.

## 1. Introduction

Melanin is a broad term for a group of natural pigments found in bacteria, fungi, plants, and animals. It is a heterogeneous, polyphenol-like biopolymer with a complex structure, and its color varies from yellow to black [1,2,3,4,5]. The color of mammalian skin and hair is determined by several factors, the most important one being the degree and distribution of melanin pigmentation [3,4,5,6]. Melanin is produced in the skin and hair [7,8]. Its role is to protect the skin from UV damage by absorbing UV light and removing reactive oxygen species [9,10]. However, excess production of melanin due to prolonged exposure to sunlight causes dermatologic disorders such as melasma, freckles, post-inflammatory melanoderma, and solar lentigines [9,11,12,13]. Melanin is secreted from melanocytes distributed in the basal layer of the dermis. Melanocytes are known to be stimulated by various factors including UV radiation [14], POMC-derived *α*-melanocyte-stimulating hormone (*α*-MSH), and other neuropeptides [7,15,16,17], and phosphodiesterase inhibitors, such as theophylline [18]. Stimulation by these factors increases melanin production using l-tyrosine and l-3,4-dihydroxyphenylalanine (l-DOPA) as substrates through various mechanisms of action [7,19]. In addition to being substrates, l-tyrosine and l-DOPA act as bioregulatory agents [19].

In our previous investigation of compounds from several natural resources possessing melanogenesis inhibitory activity, we reported that dimeric pyrrolidinoindoline- [20], aporphine- [21,22], benzylisoquinoline- [22], and phenanthridine-type [23] alkaloids, as well as phenylethanoid glycosides [23], methoxyflavones [24], phenylpropanoids [25], neolignans [25], and diterpenes [26,27] exhibited significant positive effects against theophylline-stimulated melanogenesis in B16 melanoma 4A5 cells. As a continuing study on melanogenesis inhibitors from naturally occurring compounds and their related analogs, we focus on l-ascorbic acid (AsA), one of the most recognized sugar acids, and its highly stable derivatives. AsA has multifunctional benefits on parameters affecting skin aesthetics, such as the reduction in oxidative stress and increase in collagen production. Among them, the prevention and improvement in skin pigmentation by AsA and its derivatives are, collectively, one of the most important benefits for people [28,29,30,31,32,33,34,35,36]. However, it is well known that AsA is unstable in formulations, and has a low ability to penetrate the skin due to its hydrophilicity. In addition, existing water-soluble AsA derivatives, which were developed to improve its stability [30,32], have low skin penetration. To limit these disadvantages, we recently synthesized several alkylglyceryl-AsA derivatives (**1**–**28**) by introducing a glycerol group and an alkyl group to the 2,3-enediol positions in AsA [37]. In the present study, we examine the inhibitory effects of these amphiphilic AsA derivatives (**1**–**28**) on melanogenesis in theophylline-stimulated murine B16 melanoma 4A5 cells.

## 2. Results and Discussion

### 2.1. Syntheses of Alkylglyceryl AsA Derivatives *(**1–28**)*

**Figure 1:**
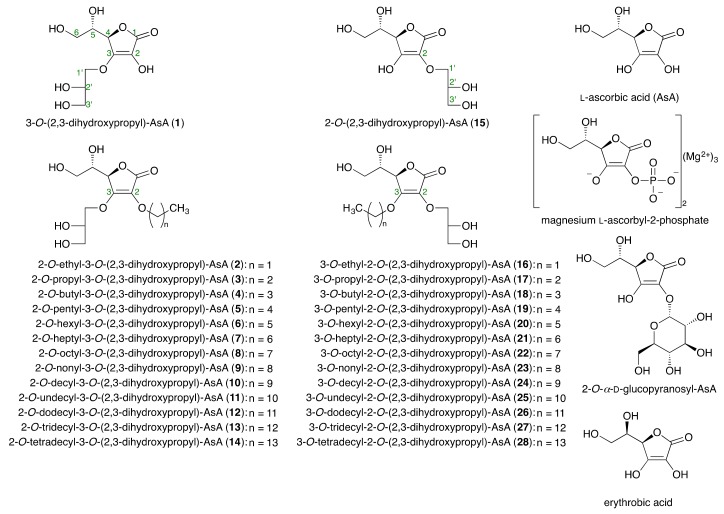
Structures of AsA derivatives (**1**–**28**). AsA: l-ascorbic acid.

As shown in Figure 1, a variety of alkylglyceryl-AsA derivatives were synthesized as described previously [37]: 3-*O*-(2,3-dihydroxypropyl)-AsA (**1**), 3-*O*-(2,3-dihydroxypropyl)-2-*O*-ethyl-AsA (**2**), 3-*O*-(2,3-dihydroxypropyl)-2-*O*-propyl-AsA (**3**), 2-*O*-butyl-3-*O*-(2,3-dihydroxypropyl)-AsA (**4**), 3-*O*-(2,3-dihydroxypropyl)-2-*O*-pentyl-AsA (**5**), 3-*O*-(2,3-dihydroxypropyl)-2-*O*-hexyl-AsA (**6**), 3-*O*-(2,3-dihydroxypropyl)-2-*O*-heptyl-AsA (**7**), 3-*O*-(2,3-dihydroxypropyl)-2-*O*-octyl-AsA (**8**), 3-*O*-(2,3-dihydroxypropyl)-2-*O*-nonyl-AsA (**9**), 2-*O*-decyl-3-*O*-(2,3-dihydroxypropyl)-AsA (**10**), 3-*O*-(2,3-dihydroxypropyl)-2-*O*-undecyl-AsA (**11**), 3-*O*-(2,3-dihydroxypropyl)-2-*O*-dodecyl-AsA (**12**), 3-*O*-(2,3-dihydroxypropyl)-2-*O*-tridecyl-AsA (**13**), 3-*O*-(2,3-dihydroxypropyl)-2-*O*-tetradecyl-AsA (**14**), 2-*O*-(2,3-dihydroxypropyl)-AsA (**15**), 2-*O*-(2,3-dihydroxypropyl)-3-*O*-ethyl-AsA (**16**), 2-*O*-(2,3-dihydroxypropyl)-3-*O*-propyl-AsA (**17**), 3-*O*-butyl-2-*O*-(2,3-dihydroxypropyl)-AsA (**18**), 2-*O*-(2,3-dihydroxypropyl)-3-*O*-pentyl-AsA (**19**), 2-*O*-(2,3-dihydroxypropyl)-3-*O*-hexyl-AsA (**20**), 2-*O*-(2,3-dihydroxypropyl)-3-*O*-heptyl-AsA (**21**), 2-*O*-(2,3-dihydroxypropyl)-3-*O*-octyl-AsA (**22**), 2-*O*-(2,3-dihydroxypropyl)-3-*O*-nonyl-AsA (**23**), 3-*O*-decyl-2-*O*-(2,3-dihydroxypropyl)-AsA (**24**), 2-*O*-(2,3-dihydroxypropyl)-3-*O*-undecyl-AsA (**25**), 2-*O*-(2,3-dihydroxypropyl)-3-*O*-dodecyl-AsA (**26**), 2-*O*-(2,3-dihydroxypropyl)-3-*O*-tridecyl-AsA (**27**), and 2-*O*-(2,3-dihydroxypropyl)-3-*O*-tetradecyl-AsA (**28**).

Considering general applications for cosmetics, these AsA derivatives (**2**–**14** and **16**–**28**) were designed to have both a hydrophilic glyceryl moiety and lipophilic alkyl chain to obtain high skin permeation and high stability in aqueous solution. Bos and Meinardi suggested that the development of new innovative compounds should be restricted to a molecular weight of less than 500 Dalton when topical dermatological therapy, percutaneous systemic therapy, or vaccination is the objective for pharmaceutical development purposes [38]. Therefore, we designed the AsA derivatives with molecular weights from 278 (**2** and **16**) to 446 (**14** and **28**) in accordance with their proposed “500 Dalton rule”. As plotted in Figure S1, excellent correlation was observed between *c*log P values and the length of the alkyl chains in these AsA derivatives (**2**–**14** and **16**–**28**) (*R* = 0.9998).

### 2.2. Effects of the Alkylglyceryl AsA Derivatives *(**1–28**)* and Commercially Available AsA Derivatives on Theophylline-Stimulated Melanogenesis Inhibitory Activity

**Figure 2:**
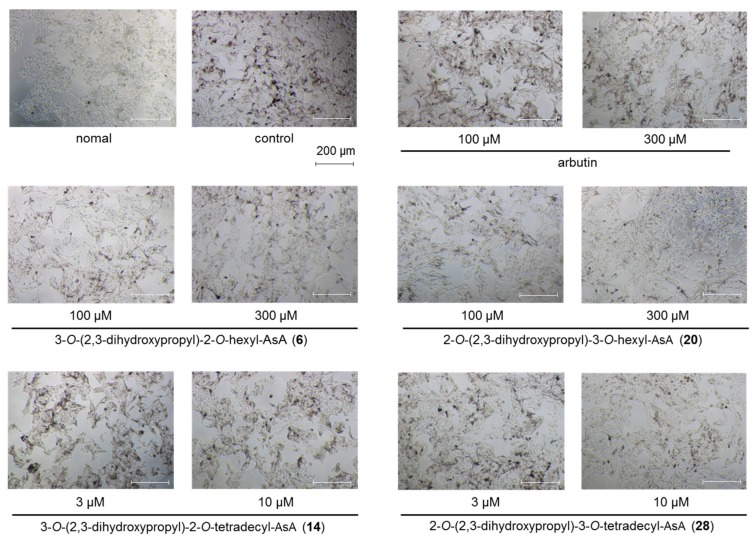
Theophylline-stimulated B16 melanoma 4A5 cells 72 h after treatment with **6** (100 µM, 300 µM), **20** (100 µM, 300 µM), **14** (3 µM, 10 µM,), or **28** (3 µM, 10 µM). The images are representative of several experiments. normal: theophylline(−); control: theophylline(+).

**Figure 3:**
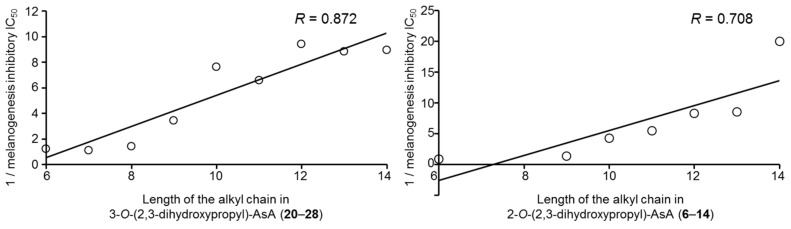
Correlation between melanogenesis inhibitory activity [1/IC_50_ values (µM)] and length of the alkyl chain in 2-*O*-alkyl-3-*O*-(2,3-dihydroxypropyl)-AsA compounds (**6**–**14**) and 3-*O*-alkyl-2-*O*-(2,3-dihydroxypropyl)-AsA compounds (**20**–**28**). AsA: l-ascorbic acid.

**Figure 4:**
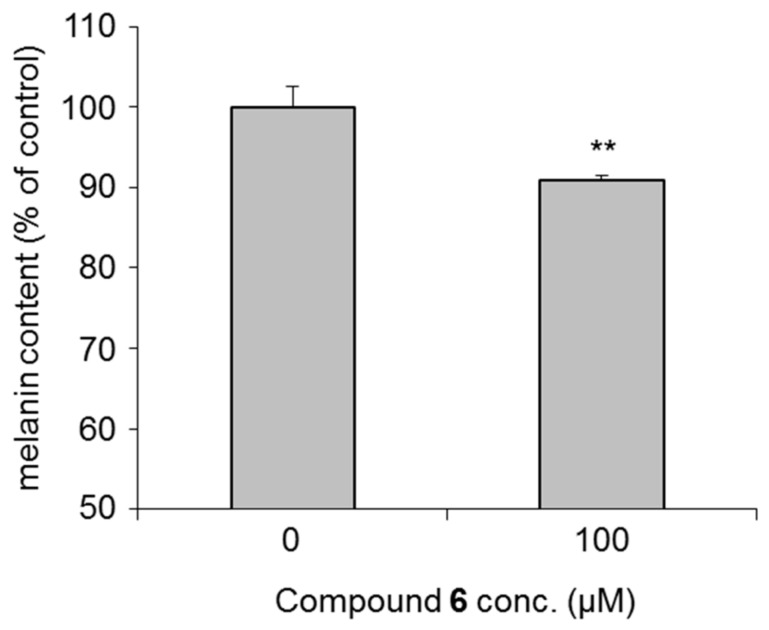
Inhibitory effect of 3-*O*-(2,3-dihydroxypropyl)-2-*O*-hexyl-AsA (**6**) on melanogenesis by normal melanocytes. Each value represents the mean ± S.D. (*n* = 3); asterisks denote significant differences from the control group, ** *p* < 0.01.

Since AsA and existing AsA derivatives are used in cosmetic materials as skin whitening and/or brightening agents [39,40], we compared their effects with those of the alkylglyceryl-AsA derivatives (**1**–**28**) on theophylline-stimulated melanogenesis in B16 melanoma 4A5 cells. As shown in Table 1, the commercially available derivatives, magnesium l-ascorbyl-2-phosphate and 2-*O*-*α*-d-glucopyranosyl-AsA, were found to significantly inhibit melanogenesis at a concentration of 3000 µM. However, these derivatives were weaker than a commercially used tyrosinase inhibitor, arbutin (IC_50_ = 830 µM) [40,41]. Among a series of 2-*O*-alkyl-3-*O*-glyceryl-AsA compounds (**1**–**14**), the derivatives having 6- to 14-carbon alkyl chains (**6**–**14**) exhibited the strongest inhibitory activities (IC_50_ = 11.1–89.1 µM). Likewise, for the 3-*O*-alkyl-2-*O*-glyceryl-AsA compounds (**15**–**28**), inhibitory activities were observed for derivatives with 4- to 14-carbon alkyl chains (**18**–**28**, IC_50_ = 5.0–473 µM). The derivative 2-*O*-(2,3-dihydroxypropyl)-3-*O*-tetradecyl-AsA (**28**, IC_50_ = 5.0 µM) exhibited the most potent inhibitory effect without notable cytotoxicity at effective concentrations. This melanogenesis inhibitory activity was found to be equivalent to that of the active metabolite of arbutin (IC_50_ = 830 µM), hydroquinone [40] (IC_50_ = 8.7 µM), and compound **28** was more than 166-fold more potent than arbutin (Figure 2). Plotting the alkyl chain length vs. the IC_50_ further revealed that the alkyl chain length is directly proportional to the inhibitory activity of the derivatives (Figure 3). Specifically, the potency of the derivative increased with increasing alkyl chain length. Compounds with longer alkyl chains (compounds **9**–**14** and **24**–**28**), however, were also found to be cytotoxic at concentration ranges similar to those required for their melanogenesis inhibitory activities. Considering the need for both safety and effectiveness for cosmetic use, the derivatives having a hexyl chain, such as 3-*O*-(2,3-dihydroxypropyl)-2-*O*-hexyl-AsA (**6**) and 2-*O*-(2,3-dihydroxypropyl)-3-*O*-hexyl-AsA (**20**), were considered to be the most valuable candidates for study due to their low toxicity and relatively high potency. Compound **6** at 100 µM significantly suppressed the inhibition of melanin production as compared with the control in normal melanocytes (Figure 4). Accordingly, for other compounds, it is necessary to determine whether the same trend is observed even in normal melanocytes.

### 2.3. Stability in Aqueous Solution

**Figure 5:**
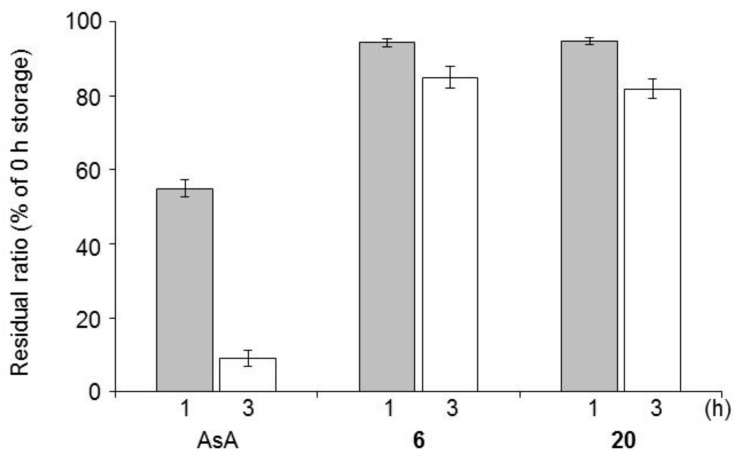
Residual ratio of **6**, **20**, and AsA in aqueous solution (20 mg/mL at 125 °C in the dark) after 1 h (gray bars) and 3 h (white bars). Each value represents the mean ± S.D. (*n* = 3); AsA: l-ascorbic acid.

We examined the stability of 3-*O*-(2,3-dihydroxypropyl)-2-*O*-hexyl-AsA (**6**) and 2-*O*-(2,3-dihydroxypropyl)-3-*O*-hexyl-AsA (**20**) in aqueous solution at elevated temperature. As presented in Figure 5, residual AsA was found to decrease rapidly with only 9% remaining after 3 h. However, more than 80% of **6** (85%) and **20** (82%) remained after 3 h of treatment.

### 2.4. Effects on Tyrosinase

**Figure 6:**
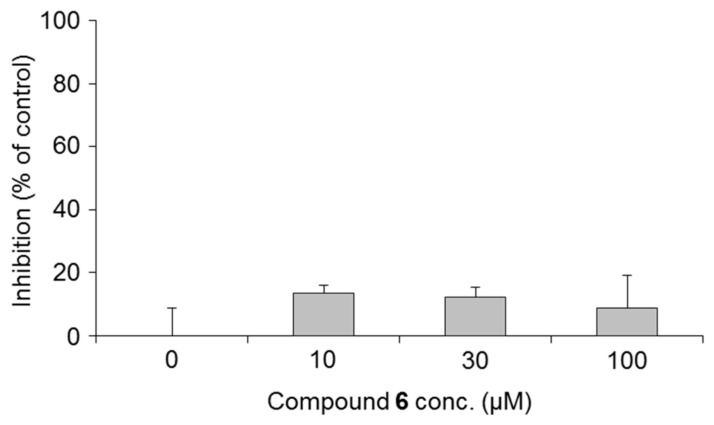
Effects of 3-*O*-(2,3-dihydroxypropyl)-2-*O*-hexyl-AsA (**6**) on the activity of mammalian tyrosinase. Each value represents the mean ± S.D. (*n* = 3).

Tyrosinase, a copper-containing enzyme, plays a key role in melanin biosynthesis, which is involved in determining the color of skin and hair [42]. It catalyzes the oxidation of both l-tyrosine to l-DOPA, and l-DOPA to dopaquinone. Dopaquinone then undergoes a chain of oxidative polymerizations to yield melanin. Tyrosinase inhibitors are clinically used for the treatment of several dermatological disorders associated with melanin hyperpigmentation [43,44]. The tyrosinase inhibitor kojic acid is commonly used as an additive in cosmetics for skin whitening and/or depigmentation [10,45]. AsA reduces *o*-quinones to *o*-diphenols. Thus, AsA inhibits melanin production by reducing back quinone structures, such as l-DOPAquinone, to l-DOPA [45,46,47,48]. Therefore, to characterize the mode of action of melanogenesis inhibitory activity of the derivatives (**6**–**14** and **20**–**28**), their effects on mushroom tyrosinase enzymatic activity were examined. As shown in Table 2, none of the AsA derivatives showed inhibitory activity when using either l-tyrosine or l-DOPA as substrates. In addition, in mammalian tyrosinase testing, compound **6** did not show activity when using l-DOPA as substrates [49], and this result was similar to that observed in the mushroom tyrosinase test (Figure 6). This suggests that tyrosinase inhibition is not involved in the mechanisms of action of these melanogenesis inhibitors.

### 2.5. Effects on Expression of Tyrosinase, TRP-1, and TRP-2 mRNA

The TRP enzyme family (tyrosinase, TRP-1, and TRP-2) catalyzes the major steps in melanin synthesis [50]. To clarify the mechanisms of action of the derivatives, we examined the effects of **6** and **20** on the expression of tyrosinase, TRP-1, and TRP-2 mRNAs in B16 melanoma 4A5 cells. As presented in Table 3, both **6** and **20** significantly downregulated the mRNA expression of tyrosinase and TRP-1 at 100 µM; **14** and **28** significantly downregulated the mRNA expression of tyrosinase and TRP-2 at 10 µM.

### 2.6. Effects on Expression of Tyrosinase Protein

**Figure 7:**
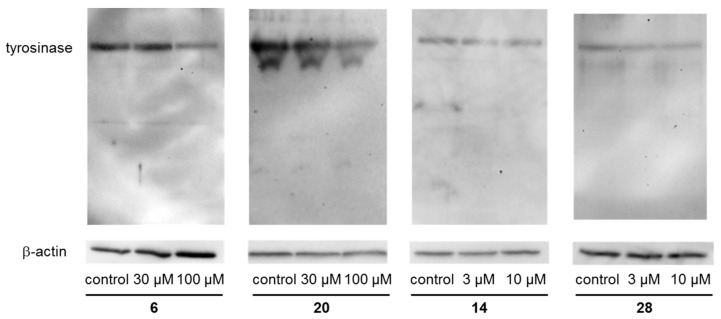
Effects of **6, 14, 20** and **28** on the expression of tyrosinase protein in B16 4A5 cells. The images are representative of several experiments.

**Figure 8:**
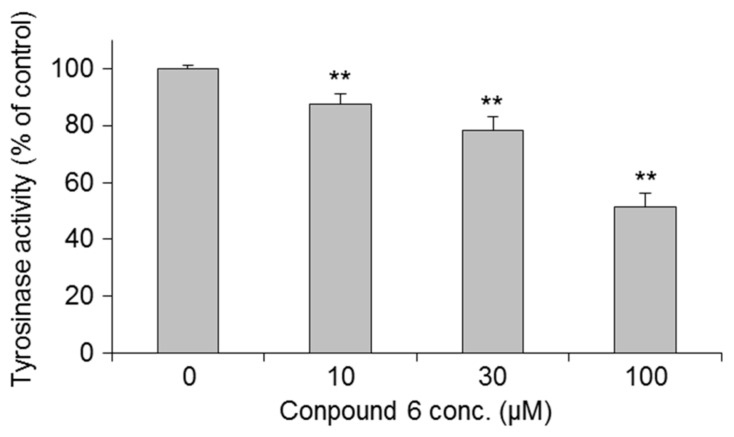
Effects of 3-*O*-(2,3-dihydroxypropyl)-2-*O*-hexyl-AsA (**6**) on tyrosinase activity in cultured cells. Each value represents the mean ± S.D (*n* = 3); asterisks denote significant differences from the control group, ** *p* < 0.01.

We next examined the effects of **6** and **20** on the expression of tyrosinase protein since it is the rate-limiting enzyme in melanin synthesis [51]. As presented in Figure 7, both **6** and **20** suppressed tyrosinase protein expression in a concentration-dependent manner. Compound **6** decreased tyrosinase activity in cultured cells when using l-DOPA as substrates (Figure 8). This suggests that tyrosinase activity in cultured cells is decreased via suppression of the expression of tyrosinase.

## 3. Materials and Methods

### 3.1. General Experimental Procedures

The following instruments were used to obtain physical data: melting points, Yanagimoto micromelting point apparatus (Yanaco New Science Inc., Kyoto, Japan); specific rotations, JASCO P-2200 digital polarimeter (JASCO Corporation, Tokyo, Japan, *l* = 5 cm); UV spectra, UV-1600 spectrometer (Shimadzu Co., Kyoto, Japan); IR spectra, IRPrestige-21 spectrometer (Shimadzu Co.); high-resolution electrospray ionization mass spectrometry (HRESIMS), Exactive Plus mass spectrometer (Thermo Fisher Scientific Inc., Waltham, MA, USA); ^1^H-NMR spectra, JNM-ECA600 (600 MHz), JNM-ECA500 (500 MHz), and JNM-ECS400 (400 MHz) spectrometers (JEOL Ltd., Tokyo, Japan); ^13^C-NMR spectra, JNM-ECA600 (150 MHz), JNM-ECA500 (125 MHz), and JNM-ECS400 (100 MHz) spectrometers (JEOL Ltd.) with tetramethylsilane as an internal standard; and HPLC detector, SPD-M20A PDA detector (Shimadzu Co.); HPLC column, Cadenza CD-C18 (Imtakt Co., Kyoto, Japan). The following experimental conditions were used for chromatography: ordinary-phase silica gel column chromatography, silica gel 60N (Kanto Chemical Co., Tokyo, Japan; 63–210 mesh, spherical, neutral); and normal-phase TLC, pre-coated TLC plates with silica gel 60F_254_ (Merck, Darmstadt, Germany; 0.25 mm), detection was carried out by spraying 2% H_3_[PMo_12_O_40_]·nH_2_O–5% aqueous H_2_SO_4_ on the plates, followed by heating. All chemicals were reagent grade, and were purchased from Wako Pure Chemical Industries, Ltd., Tokyo, Japan or Nacalai Tesque Inc., Kyoto, Japan.

### 3.2. Syntheses of Alkylglyceryl Ascorbic Acid Derivatives

The alkylglyceryl AsA derivatives (**1**–**28**) were synthesized by following general procedure: a solution of **1:15** DMSO to alkyl bromide was stirred at 100 °C for 3 h. The reaction mixture was diluted with water, and extracted with EtOAc. The organic layer was washed with brine (saturated sodium chloride in water), dried, and evaporated. The residue was purified by silica gel chromatography with CHCl_3_/MeOH/H_2_O as the mobile phase. The detailed experimental procedures were described previously [37].

3-*O*-(2,3-dihydroxypropyl)-AsA (**1**): An amorphous powder; ${\left[\alpha \right]}_{D}^{26}$ + 15.4. (*c* 3.70, MeOH); UV [MeOH, nm (log *ε*)]: 244 (3.32); IR (TlBr) *v*_max_ cm^−1^: 3275, 1759, 1693, 1335, 1045; ^1^H-NMR (600 MHz, CD_3_OD): *δ* 3.59 (2H, m, H_2_-3′), 3.66 (2H, m, H_2_-6), 3.89 (1H, m, H-5), 3.92 (1H, m, H-2′), [4.45 (dd, *J* = 6.5, 11.0 Hz)/4.49 (dd, *J* = 6.5, 11.0 Hz), 4.59 (dd, *J* = 3.8, 11.0 Hz)/4.62 (dd, *J* = 3.8, 11.0 Hz), H_2_-1′], 4.82 (1H, d, *J* = 1.7 Hz, H-4); ^13^C-NMR (150 MHz, CD_3_OD): *δ*_C_ 63.4 (t, C-6), 63.7 (t, C-3′), 70.56/70.61 (d, C-5), 71.79/71.89 (d, C-2′), 73.4/73.6 (t, C-1′), 76.9 (d, C-4), 121.17/121.24 (s, C-2), 151.84/151.88 (s, C-3), 173.04/173.07 (s, C-1); HRESIMS *m*/*z*: 273.0577 [M + Na]^+^ (calcd for C_9_H_14_O_8_Na, 273.0581).

3-*O*-(2,3-dihydroxypropyl)-2-*O*-ethyl-AsA (**2**): An amorphous powder; ${\left[\alpha \right]}_{D}^{26}$ + 37.0 (*c* 0.32, MeOH); UV [MeOH, nm (log *ε*)]: 236 (3.91); IR (ATR) *v*_max_ cm^−1^: 3316, 2934, 2889, 1748, 1667, 1321, 1169, 1111, 1026; ^1^H-NMR (400 MHz, CD_3_OD): *δ* 1.31 (3H, t, *J* = 7.4 Hz, H_3_-2″), 3.60 (2H, brd, *J* = 5.5 Hz, H_2_-3′), 3.65 (2H, dd-like, *J* = 1.8, 5.5 Hz, H_2_-6), 3.90 (2H, m, H-5, 2′), 4.09 (2H, m, H_2_-1″), [4.47/4.48 (1H, dd, *J* = 6.4, 10.5 Hz), 4.59/4.60 (1H, dd, *J* = 3.6, 10.5 Hz), H_2_-1′], 4.88 (1H, brs, H-4); ^13^C-NMR (100 MHz, CD_3_OD): *δ*_C_ 15.5 (q, C-2″), 63.2 (t, C-6), 63.5 (t, C-3′), 69.1 (t, C-1″), 70.46/70.50 (d, C-5), 71.49/71.55 (d, C-2′), 73.97/74.05 (t, C-1′), 76.7 (d, C-4), 123.87/123.91 (s, C-2), 159.53/159.58 (s, C-3), 172.3 (s, C-1); HRESIMS *m*/*z*: 301.0883 [M + Na]^+^ (calcd for C_11_H_18_O_8_Na, 301.0894).

3-*O*-(2,3-dihydroxypropyl)-2-*O*-propyl-AsA (**3**): An amorphous powder; ${\left[\alpha \right]}_{D}^{26}$ + 41.7 (*c* 0.33, MeOH); UV [MeOH, nm (log *ε*)]: 235 (3.94); IR (ATR) *v*_max_ cm^−1^: 3325, 2940, 2880, 1748, 1669, 1323, 1165, 1113, 1040; ^1^H-NMR (400 MHz, CD_3_OD): *δ* 0.99 (3H, t, *J* = 7.4 Hz, H_3_-3″), 1.72 (2H, m, H_2_-2″), 3.60 (2H, brd, *J* = 6.0 Hz, H_2_-3′), 3.65 (2H, dd-like, *J* = 1.4, 6.4 Hz, H_2_-6), 3.91 (2H, m, H-5, 2′), 3.99 (2H, m, H-1″), [4.47/4.48 (1H, dd, *J* = 6.4, 10.5 Hz), 4.58/4.59 (1H, dd, *J* = 4.1, 11.0 Hz), H_2_-1′], 4.87 (1H, brs, H-4); ^13^C-NMR (100 MHz, CD_3_OD): *δ*_C_ 10.7 (q, C-3″), 24.0 (t, C-2″), 63.2 (t, C-6), 63.5 (t, C-3′), 69.1 (t, C-1″), 70.49/70.53 (d, C-5), 71.5/71.6 (d, C-2′), 74.0/74.1 (t, C-1′), 75.55/75.58 (t, C-1′), 76.7 (d, C-4), 123.24/123.26 (s, C-2), 159.27/159.30 (s, C-3), 172.3 (s, C-1); HRESIMS *m*/*z*: 315.1046 [M + Na]^+^ (calcd for C_12_H_20_O_8_Na, 315.1050).

2-*O*-butyl-3-*O*-(2,3-dihydroxypropyl)-AsA (**4**): An amorphous powder; ${\left[\alpha \right]}_{D}^{26}$ + 47.4 (*c* 0.34, MeOH); UV [MeOH, nm (log *ε*)]: 236 (3.93); IR (TlBr) *v*_max_ cm^−1^:3393, 1749, 1674, 1331, 1167, 1117, 1049; ^1^H-NMR (500 MHz, CD_3_OD): *δ* 0.96 (3H, t, *J* = 7.5 Hz, H_3_-4″), 1.45 (2H, m, H_2_-3″), 1.68 (2H, m, H_2_-2″), 3.60 (2H, brd, *J* = 5.8 Hz, H_2_-3′), 3.65 (2H, m, H_2_-6), 3.90 (1H, m, H-5), 3.91 (1H, m, H-8), 4.03 (1H, m, H-1″), [4.47 (dd, *J* = 6.0, 10.6 Hz)/4.48 (dd, *J* = 6.3, 10.7 Hz), 4.57 (dd, *J* = 3.8, 10.7 Hz)/4.59 (dd, *J* = 4.0, 10.6 Hz), H_2_-1′], [4.858 (d, *J* = 1.5 Hz)/4.859 (d, *J* = 1.5 Hz), H-4]; ^13^C-NMR (125 MHz, CD_3_OD): *δ*_C_ 14.1 (q, C-4″), 20.1 (t, C-3″), 32.9 (t, C-2″), 63.2 (t, C-6), 63.56/63.59 (t, C-3′), 70.5/70.6 (d, C-5), 71.5/71.6 (d, C-2′), 73.69/73.72 (d, C-1′), 74.0/74.1 (t, C-1″), 76.7 (d, C-4), 123.28/123.30 (s, C-2), 159.23/159.25 (s, C-3), 172.2 (s, C-1); HRESIMS *m*/*z*: 329.1203 [M + Na]^+^ (calcd for C_13_H_22_O_8_Na, 329.1207).

3-*O*-(2,3-dihydroxypropyl)-2-*O*-pentyl-AsA (**5**): An amorphous powder; ${\left[\alpha \right]}_{D}^{26}$ + 40.9 (*c* 0.32, MeOH); UV [MeOH, nm (log *ε*)]: 235 (3.98); IR (ATR) *v*_max_ cm^−1^: 3304, 2934, 2874, 1750, 1670, 1321, 1165, 1115, 1032; ^1^H-NMR (400 MHz, CD_3_OD): *δ* 0.93 (3H, t, *J* = 6.8 Hz, H_3_-5″), 1.39 (4H, m, H_2_-3″, 4″), 1.70 (2H, m, H_2_-2″), 3.60 (2H, brd, *J* = 5.5 Hz, H_2_-3′), 3.65 (2H, dd-like, *J* = 1.4, 6.4 Hz, H_2_-6), 3.91 (2H, m, H-5, 2′), 4.02 (1H, m, H-1″), [4.47/4.48 (1H, dd, *J* = 6.4, 10.6 Hz, *J* = 6.0, 10.6 Hz), 4.58/4.59 (1H, dd, *J* = 4.1, 10.6 Hz), H_2_-1′], 4.86 (1H, brs, H-4); ^13^C-NMR (100 MHz, CD_3_OD): *δ*_C_ 14.4 (q, C-5″), 23.5 (t, C-4″), 29.1 (t, C-3″), 30.5 (t, C-2″), 63.2 (t, C-6), 63.5/63.6 (t, C-3′), 70.48/70.52 (d, C-5), 71.5/71.6 (d, C-2′), 74.0/74.1 (t, C-1′, 1″), 76.7 (d, C-4), 123.2/123.3 (s, C-2), 159.2/159.3 (s, C-3), 172.3 (s, C-1); HRESIMS *m*/*z*: 343.1357 [M + Na]^+^ (calcd for C_14_H_24_O_8_Na, 343.1363).

3-*O*-(2,3-dihydroxypropyl)-2-*O*-hexyl-AsA (**6**): An amorphous powder; ${\left[\alpha \right]}_{D}^{26}$ + 42.2 (*c* 0.32, MeOH); UV [MeOH, nm (log *ε*)]: 236 (3.90); IR (TlBr) *v*_max_ cm^−1^:3379, 2934, 1751, 1674, 1330, 1167, 1117, 1051; ^1^H-NMR (400 MHz, CD_3_OD): *δ* 0.91 (3H, t, *J* = 6.9 Hz, H_3_-6″), 1.33(4H, m, H_2_-4″, 5″), 1.41 (2H, m, H_2_-3″), 1.70 (2H, m, H_2_-2″), 3.60 (2H, brd, *J* = 5.5 Hz, H_2_-3′), 3.65 (2H, dd-like, H_2_-6), 3.90 (2H, m, H-5, 2′), 4.03 (2H, m, H-1″), [4.47/4.48 (1H, dd, *J* = 6.4, 10.6 Hz/*J* = 6.0, 10.6 Hz), 4.57/4.59 (1H, dd, *J* = 4.1, 10.7 Hz), H_2_-1′], 4.86 (1H, brs, H-4); ^13^C-NMR (100 MHz, CD_3_OD): *δ*_C_ 14.4 (q, C-6″), 23.7 (t, C-5″), 26.6 (t, C-3″), 30.7 (t, C-2″), 32.7 (t, C-4″), 63.2 (t, C-6), 63.6 (t, C-3′), 70.5 (d, C-5), 71.5/71.6 (d, C-2′), 74.01/74.04/74.09 (t, C-1′, 1″), 76.7 (d, C-4), 123.2/123.3 (s, C-2), 159.2/159.3 (s, C-3), 172.3 (s, C-1); HRESIMS *m*/*z*: 357.1519 [M + Na]^+^ (calcd for C_15_H_26_O_8_Na, 357.1520).

3-*O*-(2,3-dihydroxypropyl)-2-*O*-heptyl-AsA (**7**): An amorphous powder; ${\left[\alpha \right]}_{D}^{26}$ + 29.8 (*c* 0.35, MeOH); UV [MeOH, nm (log *ε*)]: 236 (3.87); IR (TlBr) *v*_max_ cm^−1^:3389, 2932, 2507, 1751, 1674, 1331, 1169, 1119, 1051; ^1^H-NMR (400 MHz, CD_3_OD): *δ* 0.90 (3H, t, *J* = 6.4 Hz, H_3_-7″), 1.34 (6H, m, H_2_-4″, 5″, 6″), 1.42 (2H, m, H_2_-3″), 1.70 (2H, m, H_2_-2″), 3.60 (2H, brd, *J* = 5.5 Hz, H_2_-3′), 3.65 (2H, dd-like, *J* = 1.8, 6.4 Hz, H_2_-6), 3.90 (1H, m, H-5, 2′), 4.04 (1H, m, H_2_-1″), [4.47 (1H, dd, *J* = 6.4, 10.6 Hz), 4.57/4.59 (dd, *J* = 4.1, 10.6 Hz), H_2_-1′], 4.86 (1H, brd, *J* = 2.3 Hz), H-4]; ^13^C-NMR (100 MHz, CD_3_OD): *δ*_C_ 14.4 (q, C-7′), 23.7 (t, C-6′), 26.9/30.2/30.8/33.0 (t, C-2′, 3′, 4′, 5′), 63.2 (t, C-6), 63.5/63.6 (t, C-3′), 70.49/70.52 (d, C-5), 71.52/71.59 (d, C-2′), 74.0/74.1 (t, C-1′, 1″), 76.7 (d, C-4), 123.2/123.3 (s, C-2), 159.2/159.3 (s, C-3), 172.3 (s, C-1); HRESIMS *m*/*z*: 371.1673 [M + Na]^+^ (calcd for C_16_H_28_O_8_Na, 371.1676).

3-*O*-(2,3-dihydroxypropyl)-2-*O*-octyl-AsA (**8**): An amorphous powder; ${\left[\alpha \right]}_{D}^{26}$ + 32.6 (*c* 0.32, MeOH); UV [MeOH, nm (log *ε*)]: 236 (3.91); IR (KBr) *v*_max_ cm^−1^:3368, 2855, 1751, 1676, 1339, 1169, 1115, 1071; ^1^H-NMR (400 MHz, CD_3_OD): *δ* 0.90 (3H, t, *J* = 6.9 Hz, H_3_-8″), 1.31 (10H, m, H_2_-4″, 5″, 6″, 7″), 1.42 (2H, m, H_2_-3″), 1.70 (2H, m, H_2_-2″), 3.59 (2H, brd, *J* = 6.0 Hz, H_2_-3′), 3.65 (2H, dd-like, *J* = 1.8, 6.4 Hz, H_2_-6), 3.90 (2H, m, H-5, 2′), 4.03 (1H, m, H_2_-1″), [4.47/4.48 (1H, dd, *J* = 6.4, 10.6 Hz), 4.58/4.59 (1H, dd, *J* = 4.6, 10.6 Hz/*J* = 4.1, 10.6 Hz), H_2_-1′], 4.86 (d, *J* = 1.8 Hz, H-4); ^13^C-NMR (100 MHz, CD_3_OD): *δ*_C_ 14.5 (q, C-8″), 23.7 (t, C-7″), 27.0/30.4/30.5/30.8/33.0 (t, C-2′, 3′, 4′, 5′, 6′), 63.2 (t, C-6), 63.51/63.55 (t, C-3′), 70.49/70.52 (d, C-5), 71.52/71.59 (d, C-2′), 74.02/74.09 (t, C-1′, 1″), 76.7 (d, C-4), 123.2/123.3 (s, C-2), 159.26/159.29 (s, C-3), 172.3 (s, C-1); HRESIMS *m*/*z*: 385.1829 [M + Na]^+^ (calcd for C_17_H_30_O_8_Na, 385.1833).

3-*O*-(2,3-dihydroxypropyl)-2-*O*-nonyl-AsA (**9**): An amorphous powder; ${\left[\alpha \right]}_{D}^{26}$ + 33.3 (*c* 0.31, MeOH); UV [MeOH, nm (log *ε*)]: 236 (3.93); IR (KBr) *v*_max_ cm^−1^:3327, 2924, 2853, 1761, 1684, 1333, 1169, 1117, 1046; ^1^H-NMR (400 MHz, CD_3_OD): *δ* 0.89 (3H, t, *J* = 6.8 Hz, H-9″), 1.30 (10H, m, H_2_-4″, 5″, 6″, 7″, 8″), 1.42 (2H, m, H_2_-3″), 1.70 (2H, m, H_2_-2″), 3.60 (2H, brd, *J* = 5.5 Hz, H_2_-3′), 3.65 (2H, dd-like, *J* = 1.4, 6.4 Hz, H_2_-6), 3.91 (2H, m, H-5, 2′), 4.03 (1H, m, H_2_-1′), [4.47/4.48 (1H, dd, *J* = 6.4, 10.6 Hz), 4.58/4.59 (1H, dd, *J* = 4.6, 10.6 Hz, *J* = 4.1, 10.6 Hz), H_2_-1′], 4.86 (d, *J* = 0.9 Hz, H-4) ; ^13^C-NMR (100 MHz, CD_3_OD) ; *δ*_C_ 14.5 (q, C-9″), 23.7 (t, C-8″), 27.0/30.4/30.5/30.7/30.8/33.1 (t, C-2″, 3″, 4″, 5″, 6″, 7″), 63.2 (t, C-6), 63.5/63.6 (t, C-3′), 70.48/70.52 (d, C-5), 71.5/71.6 (d, C-2′), 74.01/74.05/74.09 (t, C-1′, 1″), 76.7 (d, C-4), 123.2/123.3 (s, C-2), 159.2/159.3 (s, C-3), 172.3 (s, C-1); HRESIMS *m*/*z*: 399.1988 [M + Na]^+^ (calcd for C_18_H_32_O_8_Na, 399.1989).

2-*O*-decyl-3-*O*-(2,3-dihydroxypropyl)-AsA (**10**): An amorphous powder; ${\left[\alpha \right]}_{D}^{26}$ + 31.4 (*c* 0.30, MeOH); UV [MeOH, nm (log *ε*)]: 236 (3.88); IR (KBr) *v*_max_ cm^−1^:3317, 2959, 2922, 2924, 2849, 1759, 1682, 1331, 1165, 1113, 1043; ^1^H-NMR (600 MHz, CD_3_OD): *δ* 0.89 (3H, t, *J* = 7.2 Hz, H_3_-10″), 1.30 (12H, brs, H_2_-4″, 5″, 6″, 7″, 8″,9″), 1.41 (2H, m, H_2_-3″), 1.69 (2H, m, H_2_-2″), 3.60 (2H, brd, *J* = 5.6 Hz, H_2_-3′), 3.65 (2H, m, H_2_-6), 3.90 (1H, m, H-5), 3.91 (1H, m, H-2′), 4.02 (1H, m, H_2_-1″), [4.47 (dd, *J* = 6.1, 10.7 Hz)/4.48 (dd, *J* = 6.2, 10.7 Hz), 4.57 (dd, *J* = 3.8, 10.7 Hz)/4.59 (dd, *J* = 3.9, 10.7 Hz), H_2_-1′], [4.860 (d, *J* = 1.1 Hz)/4.862 (d, *J* = 1.5 Hz), H-4]: ^13^C-NMR (150 MHz, CD_3_OD) ; *δ*_C_ 14.4 (q, C-10″), 23.7 (t, C-9″), 26.9 (t, C-3″), 30.48 (t, C-2″), 30.44/30.48/30.7/30.8 (t, C-4″, 5″, 6″, 7″), 33.1 (t, C-8″), 63.2 (t, C-6), 63.5/63.6 (t, C-3′), 70.5/70.6 (d, C-5), 71.5/71.6 (d, C-2′), 74.02/74.05 (t, C-1′), 74.11 (t, C-1″), 76.7 (d, C-4), 123.26/123.29 (s, C-2), 159.24/159.27 (s, C-3), 172.2 (s, C-1); HRESIMS *m*/*z*: 413.2145 [M + Na]^+^ (calcd for C1_9_H_34_O_8_Na, 413.2146).

3-*O*-(2,3-dihydroxypropyl)-2-*O*-undecyl-AsA (**11**): An amorphous powder; ${\left[\alpha \right]}_{D}^{26}$ + 34.7 (*c* 0.34, MeOH); UV [MeOH, nm (log *ε*)]: 236 (3.88); IR (KBr) *v*_max_ cm^−1^:3300, 2916, 2851, 1761, 1684, 1329, 1171, 1119, 1063, 1030; ^1^H-NMR (600 MHz, CD_3_OD): *δ* 0.89 (3H, t, *J* = 6.9 Hz, H_3_-11″), 1.29 (14H, brs, H_2_-4″, 5″, 6″, 7″, 8″, 9″, 10″), 1.41 (2H, m, H_2_-3″), 1.70 (2H, m, H_2_-2″), 3.60 (2H, brd, *J* = 5.8 Hz, H_2_-3″), 3.65 (2H, m, H_2_-6), 3.90 (1H, m, H-5), 3.91 (1H, m, H-2′), 4.02 (1H, m, H_2_-1″), [4.47 (dd, *J* = 6.3, 10.6 Hz)/4.48 (dd, *J* = 6.3, 10.6 Hz), 4.57 (dd, *J* = 4.0, 10.6 Hz)/4.59 (dd, *J* = 4.0, 10.6 Hz), H_2_-1′], [4.860 (d, *J* = 1.8 Hz)/4.861 (d, *J* = 1.8 Hz), H-4]: ^13^C-NMR (150 MHz, CD_3_OD); *δ*_C_ 14.4 (q, C-11″), 23.7 (t, C-10″), 26.9 (t, C-3″), 30.70 (t, C-2″), 30.47/30.49/30.70/30.74/30.8 (t, C-4″, 5″, 6″, 7″, 8″), 33.1 (t, C-9″), 63.2 (t, C-6), 63.5/63.6 (t, C-3′), 70.5/70.6 (d, C-5), 71.5/71.6 (d, C-2′), 74.02/74.05 (t, C-1′), 74.11 (t, C-1″), 76.7 (d, C-4), 123.27/123.29 (s, C-2), 159.2/159.3 (s, C-3), 172.4 (s, C-1); HRESIMS *m*/*z*: 427.2299 [M + Na]^+^ (calcd for C_20_H_36_O_8_Na, 427.2302).

3-*O*-(2,3-dihydroxypropyl)-2-*O*-dodecyl-AsA (**12**): An amorphous powder; ${\left[\alpha \right]}_{D}^{26}$ + 38.4 (*c* 0.31, MeOH); UV [MeOH, nm (log *ε*)]: 236 (3.86); IR (KBr) *v*_max_ cm^−1^:3422, 2918, 2851, 1749, 1676, 1319, 1115, 1070; ^1^H-NMR (500 MHz, CD_3_OD): *δ* 0.89 (3H, t, *J* = 6.6 Hz, H_3_-12″), 1.29 (16H, brs, H_2_-4″, 5″, 6″, 7″, 8″, 9″, 10″, 11″), 1.41 (2H, m, H_2_-3″), 1.70 (2H, m, H_2_-2″), 3.60 (2H, brd, *J* = 5.7 Hz, H_2_-3′), 3.65 (2H, m, H_2_-6), 3.90 (1H, m, H-5), 3.91 (1H, m, H-2′), 4.02 (1H, m, H_2_-1″), [4.47 (dd, *J* = 6.4, 10.7 Hz)/4.48 (dd, *J* = 6.4, 10.7 Hz), 4.57 (dd, *J* = 4.0, 10.7 Hz)/4.59 (dd, *J* = 4.0, 10.7 Hz), H_2_-7], 4.86 (1H, m, *J* = 6.6 Hz, H-2′): ^13^C-NMR (125 MHz, CD_3_OD) ; *δ*_C_ 14.4 (q, C-12″), 23.7 (t, C-11″), 27.0 (t, C-3″), 30.5 (t, C-4″), 30.76 (t, C-2″), 30.72/30.76/30.79/30.81 (t, C-5″, 6″, 7″, 8″, 9″, 10″), 33.1 (t, C-10″), 63.2 (t, C-6), 63.56/63.58 (t, C-3′), 70.5/70.6 (d, C-5), 71.5/71.6 (d, C-2′), 74.0 (t, C-7), 74.1 (t, C-1″), 76.7 (d, C-4), 123.27/123.29 (s, C-2), 159.2/159.3 (s, C-3), 172.2 (s, C-1); HRESIMS *m*/*z*: 441.2457 [M + Na]^+^ (calcd for C_21_H_38_O_8_Na, 441.2459).

3-*O*-(2,3-dihydroxypropyl)-2-*O*-tridecyl-AsA (**13**): An amorphous powder; ${\left[\alpha \right]}_{D}^{26}$ + 35.8 (*c* 0.30, MeOH); UV [MeOH, nm (log *ε*)]: 231 (4.02); IR (KBr) *v*_max_ cm^−1^:3289, 2916, 2849, 1761, 1684, 1329, 1119, 1171, 1119, 1063, 1030; ^1^H-NMR (500 MHz, CD_3_OD): *δ* 0.89 (3H, t, *J* = 6.9 Hz, H_3_-13″), 1.28 (18H, brs, H_2_-4″, 5″, 6″, 7″, 8″, 9″, 10″, 11″, 12″), 1.41 (2H, m, H_2_-3″), 1.70 (2H, m, H_2_-2″), 3.60 (2H, brd, *J* = 5.8 Hz, H_2_-3′), 3.65 (2H, m, H_2_-6), 3.90 (1H, m, H-5), 3.91 (1H, m, H-2′), 4.02 (1H, m, H_2_-1″), [4.47 (dd, *J* = 6.3, 10.7 Hz)/4.48 (dd, *J* = 6.3, 10.7 Hz), 4.57 (dd, *J* = 4.0, 10.7 Hz)/4.59 (dd, *J* = 4.0, 10.7 Hz), H_2_-1′], [4.859 (d, *J* = 1.8 Hz)/4.861 (d, *J* = 1.8 Hz), H-4]: ^13^C-NMR (125 MHz, CD_3_OD) ; *δ*_C_ 14.4 (q, C-13″), 23.7 (t, C-12″), 27.0 (t, C-3″), 30.81 (t, C-2″), 30.48/30.50/30.71/30.78/30.81 (t, C-4″, 5″, 6″, 7″, 8″, 9″, 10″, 11″), 33.1 (t, C-12″), 63.2 (t, C-6), 63.56/63.58 (t, C-3′), 70.5/70.6 (d, C-5), 71.5/71.6 (d, C-2′), 74.0 (t, C-1′), 74.1 (t, C-1″), 76.7 (d, C-4), 123.27/123.29 (s, C-2), 159.2/159.3 (s, C-3), 172.2 (s, C-1); HRESIMS *m*/*z*: 455.2612 [M + Na]^+^ (calcd for C_22_H_40_O_8_Na, 455.2615).

3-*O*-(2,3-dihydroxypropyl)-2-*O*-tetradecyl-AsA (**14**): An amorphous powder; ${\left[\alpha \right]}_{D}^{26}$ + 24.4 (*c* 0.31, MeOH); UV [MeOH, nm (log *ε*)]: 230 (3.99); IR (KBr) *v*_max_ cm^−1^:3326, 2920, 2849, 1759, 1680, 1466, 1329, 1165, 1115, 1034; ^1^H-NMR (500 MHz, CD_3_OD): *δ* 0.89 (3H, t, *J* = 7.2 Hz, H_3_-14″), 1.28 (20H, brs, H_2_-4″, 5″, 6″, 7″, 8″, 9″, 10″, 11″, 12″, 13″), 1.41 (2H, m, H_2_-3″), 1.70 (2H, m, H_2_-2″), 3.60 (2H, brd, *J* = 5.8 Hz, H_2_-3′), 3.65 (2H, m, H_2_-6), 3.90 (1H, m, H-5), 3.91 (1H, m, H-2′), 4.02 (1H, m, H_2_-1″), [4.47 (dd, *J* = 6.4, 10.7 Hz)/4.48 (dd, *J* = 6.4, 10.7 Hz), 4.57 (dd, *J* = 3.8, 10.7 Hz)/4.59 (dd, *J* = 3.8, 10.7 Hz), H_2_-1′], [4.858 (d, *J* = 1.5 Hz)/4.860 (d, *J* = 1.5 Hz), H-4]: ^13^C-NMR (125 MHz, CD_3_OD) ; *δ*_C_ 14.4 (q, C-14″), 23.7 (t, C-13″), 27.0 (t, C-3″), 30.80 (t, C-2″), 30.48/30.51/30.72/30.76/30.80 (t, C-4″, 5″, 6″, 7″, 8″, 9″, 10″, 11″), 33.1 (t, C-12″), 63.2 (t, C-6), 63.56/63.59 (t, C-3′), 70.5/70.6 (d, C-5), 71.5/71.6 (d, C-2′), 74.0 (t, C-1′), 74.1 (t, C-1″), 76.7 (d, C-4), 123.27/123.30 (s, C-2), 159.23/159.25 (s, C-3), 172.2 (s, C-1); HRESIMS *m*/*z*: 469.2769 [M + Na]^+^ (calcd for C_23_H_42_O_8_Na, 469.2772).

2-*O*-(2,3-dihydroxypropyl)-AsA (**15**): Colorless needles, mp.153.0-153.2; ${\left[\alpha \right]}_{D}^{26}$ + 55.7 (*c* 0.31, MeOH); UV [MeOH, nm (log *ε*)]: 238 (3.89); IR (KBr) *v*_max_ cm^−1^:3326, 2920, 2849, 1759, 1680, 1466, 1329, 1165, 1115, 1034; ^1^H-NMR (400 MHz, CD_3_OD): *δ* 3.61 (2H, m, H_2_-3′), 3.67 (2H, m) (2H, m, H_2_-6), 3.90 (1H, m, H-2′), 3.92 (1H, dt-like, *J* = 1.8, 6.4 Hz, H_3_-5), [4.07 (1H, dd, *J* = 4.1, 10.4 Hz)/4.09 (1H, d, *J* = 3.6, 10.4 Hz), H-1′], 4.86 (1H, d, *J* = 1.8 Hz, H-4): ^13^C-NMR (100 MHz, CD_3_OD) ; *δ*_C_ 63.3 (t, C-6), 63.7 (t, C-3′), 70.4 (d, C-5), 72.0 (d, C-2′), 74.6 (t, C-1′), 76.8 (t, C-4′), 122.2 (s, C-2), 161.6 (s, C-3), 172.9 (s, C-1); HRESIMS *m*/*z*: 273.0576 [M + Na]^+^ (calcd for C_9_H_14_O_8_Na, 273.0581).

2-*O*-(2,3-dihydroxypropyl)-3-*O*-ethyl-AsA (**16**): Pale yellow oil; ${\left[\alpha \right]}_{D}^{26}$ + 34.3 (*c* 0.33, MeOH); UV [MeOH, nm (log *ε*)]: 236 (3.86); IR (ATR) *v*_max_ cm^−1^: 3337, 2938, 2881, 1744, 1665, 1325, 1173, 1109, 1038; ^1^H-NMR (400 MHz, CD_3_OD): *δ* 1.38 (3H, t, *J* = 6.8 Hz, H_3_-2″), 3.58 (2H, m, H_2_-3′) 3.64 (2H, dd-like, *J* = 1.4, 6.4 Hz, H_2_-6), 3.87 (2H, m, H-5, 2′), [3.97 (1H, m), 4.13 (1H, m), 4.68 (2H, m, H_2_-1″), 4.83(d, *J* = 0.9 Hz, H-4): 13C-NMR (100 MHz, CD3OD) ; *δ*_C_ 15.5 (q, C-2″), 63.3 (t, C-6), 63.98/63.48 (t, C-3′), 69.6 (t, C-1″), 70.6 (d, C-5), 72.0 (d, C-2′), 75.19/75.23 (t, C-1′), 76.7 (d, C-4), 122.60/122.62 (s, C-2), 159.7 (s, C-3), 175.6 (s, C-1); HRESIMS *m*/*z*: 301.0887 [M + Na]^+^ (calcd for C_11_H_18_O_8_Na, 301.0894).

2-*O*-(2,3-dihydroxypropyl)-3-*O*-propyl-AsA (**17**): Pale yellow oil; ${\left[\alpha \right]}_{D}^{26}$ + 49.8 (*c* 0.31, MeOH); UV [MeOH, nm (log *ε*)]: 237 (3.94); IR (ATR) *v*_max_ cm^−1^: 3339, 2938, 2882, 1744, 1665, 1327, 1173, 1040; ^1^H-NMR (400 MHz, CD_3_OD): *δ* 1.01 (3H, t, *J* = 7.3 Hz, H_3_-3″), 1.78 (2H, m, H_2_-2″), 3.58 (2H, m, H_2_-3′), 3.64 (2H, dd-like, *J* = 0.9, 6.4 Hz, H_2_-6), 3.87 (2H, m, H-5, 2′), [3.96/3.97 (1H, dd, *J* = 6.4, 10.1 Hz), 4.11/4.13 (1H, dd, *J* = 4.1, 10.1 Hz), H_2_-1′], 4.50 (2H, m, H_2_-1″), 4.83(1H, d, *J* = 1.4 Hz, H-4): ^13^C-NMR (100 MHz, CD_3_OD) ; *δ*_C_ 10.3 (q, C-3″), 23.9 (t, C-2″), 63.3 (t, C-6), 64.0 (t, C-3′), 70.6 (d, C-5), 72.0 (d, C-2′), 75.1 (t, C-1″), 75.19/75.23 (t, C-1′), 76.7 (d, C-4), 122.4 (s, C-2), 159.9 (s, C-3), 172.6 (s, C-1); HRESIMS *m*/*z*: 315.1042 [M + Na]^+^ (calcd for C_12_H_20_O_8_Na, 315.1050).

3-*O*-butyl-2-*O*-(2,3-dihydroxypropyl)-AsA (**18**): Pale yellow oil; ${\left[\alpha \right]}_{D}^{26}$ + 46.6 (*c* 0.31, MeOH); UV [MeOH, nm (log *ε*)]: 237 (3.89); IR (ATR) *v*_max_ cm^−1^: 3341, 2936, 2876, 1746, 1665, 1329, 1171, 1115, 1036; ^1^H-NMR (400 MHz, CD_3_OD): *δ* 0.97 (3H, t, *J* = 7.3 Hz, H_3_-4″), 1.47 (2H, m, H_2_-3″), 1.74 (2H, m, H_2_-2″), 3.58 (2H, m, H_2_-3′), 3.64 (2H, dd-like, *J* = 0.8, 6.4 Hz, H_2_-6), 3.86 (2H, m, H-5, 2′), [3.96/3.97 (1H, dd, *J* = 6.4, 10.1 Hz), 4.11/4.13 (1H, dd, *J* = 4.1, 10.1 Hz), H_2_-1′], 4.55 (2H, m, H_2_-1″), 4.83(1H, d, *J* = 1.4 Hz, H-4): ^13^C-NMR (100 MHz, CD_3_OD) ; *δ*_C_ 14.1 (q, C-4″), 19.9 (t, C-3″), 32.7 (t, C-2″), 63.3 (t, C-6), 63.28/64.01 (t, C-3′), 70.6 (d, C-5), 71.9 (d, C-2′), 73.4 (t, C-1″), 75.2/75.3 (t, C-1′), 76.7 (d, C-4), 122.60/122.62 (s, C-2), 159.9 (s, C-3), 172.62/172.63 (s, C-1); HRESIMS *m*/*z*: 329.1200 [M + Na]^+^ (calcd for C_13_H_22_O_8_Na, 329.1207).

2-*O*-(2,3-dihydroxypropyl)-3-*O*-pentyl-AsA (**19**): Pale yellow oil; ${\left[\alpha \right]}_{D}^{26}$ + 44.4 (*c* 0.30, MeOH); UV [MeOH, nm (log *ε*)]: 236 (3.93); IR (ATR) *v*_max_ cm^−1^: 3358, 2934, 2872, 1748, 1665, 1331, 1169, 1115, 1040; ^1^H-NMR (400 MHz, CD_3_OD): *δ* 0.94 (3H, t, *J* = 6.9 Hz, H_3_-5″), 1.40 (4H, m, H_2_-3, 4″), 1.76 (2H, m, H_2_-2″), 3.58 (2H, m, H_2_-3′), 3.64 (2H, dd-like, *J* = 1.4, 6.4 Hz, H_2_-6), 3.86 (2H, m, H-5, 2′), [3.96/3.98 (1H, dd, *J* = 6.4, 10.1 Hz), 4.11/4.13 (1H, dd, *J* = 4.1, 10.1 Hz), H_2_-1′], 4.54 (2H, m, H_2_-1″), 4.84(1H, brs, H-4): ^13^C-NMR (100 MHz, CD_3_OD) ; *δ*_C_ 14.3 (q, C-5″), 23.4 (t, C-4″), 28.9 (t, C-3″), 30.3 (t, C-2″), 63.3 (t, C-6), 63.97/64.00 (t, C-3′), 70.6 (d, C-5), 72.0 (d, C-2′), 73.7 (t, C-1″), 75.21/75.25 (t, C-1′), 76.7 (d, C-4), 122.57/122.60 (s, C-2), 159.9 (s, C-3), 172.6 (s, C-1); HRESIMS *m*/*z*: 343.1357 [M + Na]^+^ (calcd for C_14_H_24_O_8_Na, 343.1363).

2-*O*-(2,3-dihydroxypropyl)-3-*O*-hexyl-AsA (**20**): Pale yellow oil; ${\left[\alpha \right]}_{D}^{26}$ + 42.5 (*c* 0.35, MeOH); UV [MeOH, nm (log *ε*)]: 237 (3.96); IR (ATR) *v*_max_ cm^−1^:3367, 2930, 2859, 1748, 1667, 1331, 1167, 1117, 1041; ^1^H-NMR (400 MHz, CD_3_OD): *δ* 0.92 (3H, t-like, *J* = 6.9 Hz, H_3_-6″), 1.34 (4H, m, H_2_-4″, 5″), 1.44 (2H, m, H_2_-3″), 1.75 (2H, m, H_2_-2″), 3.57 (2H, m, H_2_-3′) 3.63 (2H, dd-like, *J* = 1.4, 6.4 Hz, H_2_-6), 3.86 (2H, m, H-5, 2′), [3.95/3.97 (1H, dd, *J* = 6.4, 10.1 Hz), 4.10/4.12 (1H, dd, *J* = 4.1, 10.1 Hz), H_2_-1′], 4.53 (2H, m, H_2_-1″), 4.84 (1H, brs, H-4): ^13^C-NMR (100 MHz, CD_3_OD) ; *δ*_C_ 14.3 (q, C-6″), 23.6 (t, C-5″), 26.3 (t, C-4″), 30.6 (t, C-3″), 32.6 (t, C-2″) 63.3 (t, C-6), 64.00/64.03 (t, C-3′), 70.6 (d, C-5), 72.0 (d, C-2′), 73.7 (t, C-1″), 75.2/75.3 (t, C-1′), 76.7 (d, C-4), 122.60/122.63 (s, C-2), 159.9 (s, C-3), 172.6 (s, C-1); HRESIMS *m*/*z*: 357.1515 [M + Na]^+^ (calcd for C_15_H_26_O_8_Na, 357.1520).

2-*O*-(2,3-dihydroxypropyl)-3-*O*-heptyl-AsA (**21**): Pale yellow oil; ${\left[\alpha \right]}_{D}^{26}$ + 40.4 (*c* 0.34, MeOH); UV [MeOH, nm (log *ε*)]: 235 (3.91); IR (ATR) *v*_max_ cm^−1^: 3341, 2926, 2857, 1748, 1669, 1331, 1167, 1115, 1040; ^1^H-NMR (400 MHz, CD_3_OD): *δ* 0.90 (3H, t-like, *J* = 6.9 Hz, H_3_-7″), 1.36 (6H, m, H_2_-4″, 5″, 6″), 1.44 (2H, m, H_2_-3″), 1.75 (2H, m, H_2_-2″), 3.57 (2H, m, H_2_-3′) 3.64 (2H, dd-like, *J* = 1.8, 6.0 Hz, H_2_-6), 3.86 (2H, m, H-5, 2′), [3.94/3.97 (1H, dd, *J* = 6.4, 10.1 Hz), 4.11/4.13 (1H, dd, *J* = 4.1, 10.1 Hz), H_2_-1′], 4.54 (2H, m, H_2_-1″), 4.83 (1H, d, *J* = 1.8 Hz, H-4): ^13^C-NMR (100 MHz, CD_3_OD) ; *δ*_C_ 14.4 (q, C-7″), 23.7 (t, C-6″), 26.7 (t, C-5″), 30.1/30.7 (t, C-3″, 4″), 32.9 (t, C-2″), 63.3 (t, C-6), 63.97/64.01 (t, C-3′), 70.6 (d, C-5), 71.9 (d, C-2′), 73.7 (t, C-1″), 75.21/75.25 (t, C-1′), 76.7 (d, C-4), 122.58/122.60 (s, C-2), 159.9 (s, C-3), 172.6 (s, C-1); HRESIMS *m*/*z*: 371.1674 [M + Na]^+^ (calcd for C_16_H_28_O_8_Na, 371.1676).

2-*O*-(2,3-dihydroxypropyl)-3-*O*-octyl-AsA (**22**): Pale yellow oil; ${\left[\alpha \right]}_{D}^{26}$ + 42.3 (*c* 0.34, MeOH); UV [MeOH, nm (log *ε*)]: 236 (3.92); IR (ATR) *v*_max_ cm^−1^:3364, 2924, 2857, 1748, 1667, 1331, 1165, 1115, 1036; ^1^H-NMR (400 MHz, CD_3_OD): *δ* 0.90 (3H, t, *J* = 6.8 Hz, H_3_-8″), 1.32 (8H, m, H_2_-4″, 5″, 6″, 7″), 1.42 (2H, m, H_2_-3″), 1.75 (2H, m, H_2_-2″), 3.58 (2H, m, H_2_-3′) 3.63 (2H, dd-like, *J* = 1.4, 6.4 Hz, H_2_-6), 3.87 (2H, m, H-5, 2′), [3.96/3.97 (1H, dd, *J* = 6.4, 10.1 Hz), 4.11/4.12 (1H, dd, *J* = 3.9, 10.1 Hz), H_2_-1′], 4.53 (2H, m, H_2_-1″), 4.83 (1H, d, *J* = 1.4, H-4): ^13^C-NMR (100 MHz, CD_3_OD) ; *δ*_C_ 14.4 (q, C-8″), 23.7 (t, C-7″), 26.7 (t, C-6″), 30.33/30.39 (t, C-4″, 5″), 30.6 (t, C-3″), 32.9 (t, C-2″), 63.3 (t, C-6), 63.99/64.02 (t, C-3′), 70.6 (d, C-5), 72.0 (d, C-2′), 73.7 (t, C-1″), 75.2/75.3 (t, C-1′), 76.68/76.70 (d, C-4), 122.60/122.64 (s, C-2), 159.9 (s, C-3), 172.63/172.64 (s, C-1); HRESIMS *m*/*z*: 385.1827 [M + Na]^+^ (calcd for C_17_H_30_O_8_Na, 385.1833).

2-*O*-(2,3-dihydroxypropyl)-3-*O*-nonyl-AsA (**23**): Pale yellow oil; ${\left[\alpha \right]}_{D}^{26}$ + 39.1 (*c* 0.30, MeOH); UV [MeOH, nm (log *ε*)]: 237 (3.92); IR (ATR) *v*_max_ cm^−1^: 3358, 2924, 2855, 1750, 1669, 1333, 1163, 1115, 1040; ^1^H-NMR (400 MHz, CD_3_OD): *δ* 0.90 (3H, t, *J* = 7.3 Hz, H_3_-9″), 1.30 (10H, brs, H_2_-4″, 5″, 6″, 7″, 8″), 1.42 (2H, m, H_2_-3″), 1.75 (2H, m, H_2_-2″), 3.58 (2H, m, H_2_-3′) 3.64 (2H, dd-like, *J* = 1.4, 6.4 Hz, H_2_-6), 3.86 (2H, m, H-5, 2′), [3.96/3.97 (1H, dd, *J* = 6.4, 10.1 Hz), 4.11/4.13 (1H, dd, *J* = 4.1, 10.1 Hz), H_2_-1′], 4.53 (2H, m, H_2_-1″), 4.83 (1H, d, *J* = 1.4, H-4): ^13^C-NMR (100 MHz, CD_3_OD) ; *δ*_C_ 14.5 (q, C-9″), 23.7 (t, C-8″), 26.7 (t, C-7″), 30.4/30.5/30.6 (t, C-3″, 4″, 5″, 6″), 30.6 (t, C-3″), 33.1 (t, C-2″), 63.2 (t, C-6), 63.97/64.00 (t, C-3′), 70.6 (d, C-5), 71.9 (d, C-2′), 73.7 (t, C-1″), 75.2/75.3 (t, C-1′), 76.7 (d, C-4), 122.57/122.60 (s, C-2), 159.9 (s, C-3), 172.6 (s, C-1); HRESIMS *m*/*z*: 399.1984 [M + Na]^+^ (calcd for C_18_H_32_O_8_Na, 399.1989).

3-*O*-decyl-2-*O*-(2,3-dihydroxypropyl)-AsA (**24**): Pale yellow oil; ${\left[\alpha \right]}_{D}^{26}$ + 36.7 (*c* 0.33, MeOH); UV [MeOH, nm (log *ε*)]: 237 (4.02); IR (ATR) *v*_max_ cm^−1^:3364, 2924, 2855, 1749, 1666, 1333, 1165, 1119, 1030; ^1^H-NMR (400 MHz, CD_3_OD): *δ* 0.89 (3H, t, *J* = 6.9 Hz, H_3_-10″), 1.30 (12H, brs, H_2_-4″, 5″, 6″, 7″, 8″, 9″), 1.43 (2H, m, H_2_-3″), 1.75 (2H, m, H_2_-2″), 3.59 (2H, m, H_2_-3′) 3.66 (2H, dd-like, *J* = 1.4, 6.4 Hz, H_2_-6), 3.86 (2H, m, H-5, 2′), [3.96/3.97 (1H, dd, *J* = 6.4, 10.1 Hz), 4.10/4.12 (1H, dd, *J* = 4.1, 10.1 Hz), H_2_-1′], 4.53 (2H, m, H_2_-1″), 4.83 (1H, brs, H-4): ^13^C-NMR (100 MHz, CD_3_OD) ; *δ*_C_ 14.4 (q, C-10″), 23.7 (t, C-9″), 26.7 (t, C-8″), 30.4/30.7 (t, C-3″, 4″, 5″, 6″, 7″), 33.0 (t, C-2″), 63.3 (t, C-6), 64.0 (t, C-3′), 70.6 (d, C-5), 72.0 (d, C-2′), 73.7 (t, C-1″), 75.2/75.3 (t, C-1′), 76.7 (d, C-4), 122.61/122.64 (s, C-2), 159.9 (s, C-3), 172.6 (s, C-1); HRESIMS *m*/*z*: 413.2146 [M + Na]^+^ (calcd for C_19_H_34_O_8_Na, 413.2146).

2-*O*-(2,3-dihydroxypropyl)-3-*O*-undecyl-AsA (**25**): Pale yellow oil; ${\left[\alpha \right]}_{D}^{26}$ + 35.9 (*c* 0.31, MeOH); UV [MeOH, nm (log *ε*)]: 237 (3.95); IR (ATR) *v*_max_ cm^−1^: 3358, 2922, 2853, 1750, 1669, 1333, 1163, 1115, 1040; ^1^H-NMR (400 MHz, CD_3_OD): *δ* 0.89 (3H, t, *J* = 6.9 Hz, H_3_-11″), 1.29 (14H, brs, H_2_-4″, 5″, 6″, 7″, 8″, 9″, 10″), 1.44 (2H, m, H_2_-3″), 1.75 (2H, m, H_2_-2″), 3.58 (2H, m, H_2_-3′) 3.64 (2H, dd-like, *J* = 1.4, 6.4 Hz, H_2_-6), 3.86 (2H, m, H-5, 2′), [3.96/3.97 (1H, dd, *J* = 6.4, 10.1 Hz), 4.10/4.13 (1H, dd, *J* = 4.1, 10.1 Hz), H_2_-1′], 4.53 (2H, m, H_2_-1″), 4.83 (1H, d, *J* = 1.4 Hz, H-4): ^13^C-NMR (100 MHz, CD_3_OD) ; *δ*_C_ 14.4 (q, C-11″), 23.8 (t, C-10″), 26.7 (t, C-9″), 30.46/30.48/30.69/30.73/30.77 (t, C-3″, 4″, 5″, 6″, 7″, 8″), 33.1 (t, C-2″), 63.2 (t, C-6), 64.0 (t, C-3′), 70.6 (d, C-5), 71.9 (d, C-2′), 73.7 (t, C-1″), 75.2/75.3 (t, C-1′), 76.7 (d, C-4), 122.58/122.60 (s, C-2), 159.9 (s, C-3), 172.6 (s, C-1); HRESIMS *m*/*z*: 427.2301 [M + Na]^+^ (calcd for C_20_H_36_O_8_Na, 427.2302).

2-*O*-(2,3-dihydroxypropyl)-3-*O*-dodecyl-AsA (**26**): An amorphous powder; ${\left[\alpha \right]}_{D}^{26}$ + 41.7 (*c* 0.33, MeOH); UV [MeOH, nm (log *ε*)]: 237 (3.96); IR (ATR) *v*_max_ cm^−1^: 3341, 2922, 2853, 1748, 1668, 1335, 1165, 1115, 1028; ^1^H-NMR (400 MHz, CD_3_OD): *δ* 0.89 (3H, t, *J* = 6.8 Hz, H_3_-12″), 1.29 (16H, brs, H_2_-4″, 5″, 6″, 7″, 8″, 9″, 10″, 11″), 1.44 (2H, m, H_2_-3″), 1.75 (2H, m, H_2_-2″), 3.57 (2H, m, H_2_-3′) 3.64 (2H, dd-like, *J* = 1.8, 6.4 Hz, H_2_-6), 3.86 (2H, m, H-5, 2′), [3.96/3.97 (1H, dd, *J* = 6.4, 10.1 Hz), 4.11/4.13 (1H, dd, *J* = 4.1, 10.1 Hz), H_2_-1′], 4.53 (2H, m, H_2_-1″), 4.83 (1H, d, *J* = 1.4 Hz, H-4): ^13^C-NMR (100 MHz, CD_3_OD) ; *δ*_C_ 14.4 (q, C-12″), 23.7 (t, C-11″), 26.7 (t, C-10″), 30.47/30.67/30.70/30.74/30.78 (t, C-3″, 4″, 5″, 6″, 7″, 8″, 9″), 33.1 (t, C-2″), 63.3 (t, C-6), 64.00/64.03 (t, C-3′), 70.6 (d, C-5), 71.9 (d, C-2′), 73.7 (t, C-1″), 75.22/75.27 (t, C-1′), 76.7 (d, C-4), 122.61/122.64 (s, C-2), 159.9 (s, C-3), 172.6 (s, C-1); HRESIMS *m*/*z*: 441.2458 [M + Na]^+^ (calcd for C_21_H_38_O_8_Na, 441.2459).

2-*O*-(2,3-dihydroxypropyl)-3-*O*-tridecyl-AsA (**27**): An amorphous powder; ${\left[\alpha \right]}_{D}^{26}$ + 37.8 (*c* 0.30, MeOH); UV [MeOH, nm (log *ε*)]: 233 (4.02); IR (ATR) *v*_max_ cm^−1^: 3358, 2922, 2853, 1750, 1670, 1333, 1167, 1115, 1042; ^1^H-NMR (400 MHz, CD_3_OD): *δ* 0.89 (3H, t, *J* = 6.4 Hz, H_3_-13″), 1.29 (18H, brs, H_2_-4″, 5″, 6″, 7″, 8″, 9″, 10″, 11″, 12″), 1.44 (2H, m, H_2_-3″), 1.75 (2H, m, H_2_-2″), 3.58 (2H, m, H_2_-3′) 3.64 (2H, dd-like, *J* = 1.8, 6.0 Hz, H_2_-6), 3.86 (2H, m, H-5, 2′), [3.96/3.97 (1H, dd, *J* = 6.9, 10.1 Hz/6.4, 10.1Hz), 4.11/4.13 (1H, dd, *J* = 3.7, 10.1 Hz), H_2_-1′], 4.54 (2H, m, H_2_-1″), 4.83 (1H, d, *J* = 1.4 Hz, H-4): ^13^C-NMR (100 MHz, CD_3_OD) ; *δ*_C_ 14.5 (q, C-13″), 23.7 (t, C-12″), 26.7 (t, C-11″), 30.48/30.69/30.73/30.78/30.81 (t, C-3″, 4″, 5″, 6″, 7″, 8″, 9″, 10″), 33.1 (t, C-2″), 63.2 (t, C-6), 63.96/63.99 (t, C-3′), 70.6 (d, C-5), 71.9 (d, C-2′), 73.7 (t, C-1″), 75.19/75.25 (t, C-1′), 76.7 (d, C-4), 122.6 (s, C-2), 159.9 (s, C-3), 172.6 (s, C-1); HRESIMS *m*/*z*: 455.2616 [M + Na]^+^ (calcd for C_22_H_40_O_8_Na, 455.2615).

2-*O*-(2,3-dihydroxypropyl)-3-*O*-tetradecyl-AsA (**28**): An amorphous powder; ${\left[\alpha \right]}_{D}^{26}$ + 31.1 (*c* 0.30, MeOH); UV [MeOH, nm (log *ε*)]: 237 (3.93); IR (ATR) *v*_max_ cm^−1^: 3379, 2922, 2853, 1750, 1669, 1333, 1167, 1117, 1040; ^1^H-NMR (400 MHz, CD_3_OD): *δ* 0.89 (3H, t, *J* = 6.4 Hz, H_3_-14″), 1.28 (20H, brs, H_2_-4″, 5″, 6″, 7″, 8″, 9″, 10″, 11″, 12″, 13″), 1.44 (2H, m, H_2_-3″), 1.75 (2H, m, H_2_-2″), 3.58 (2H, m, H_2_-3′) 3.64 (2H, dd-like, *J* = 1.4, 6.4 Hz, H_2_-6), 3.86 (2H, m, H-5, 2′), [3.96/3.97 (1H, dd, *J* = 6.9, 10.1 Hz/6.4, 10.1Hz), 4.11/4.13 (1H, dd, *J* = 4.1, 10.1 Hz), H_2_-1′], 4.54 (2H, m, H_2_-1″), 4.83 (1H, d, *J* = 0.9 Hz, H-4): ^13^C-NMR (100 MHz, CD_3_OD); *δ*_C_ 14.5 (q, C-14″), 23.7 (t, C-13″), 26.7 (t, C-12″), 30.48/30.69/30.73/30.78/30.81 (t, C-3″, 4″, 5″, 6″, 7″, 8″, 9″, 10″, 11″), 33.1 (t, C-2″), 63.2 (t, C-6), 63.97/64.00 (t, C-3′), 70.6 (d, C-5), 71.9 (d, C-2′), 73.7 (t, C-1″), 75.20/75.25 (t, C-1′), 76.7 (d, C-4), 122.6 (s, C-2), 159.9 (s, C-3), 172.6 (s, C-1); HRESIMS *m*/*z*: 469.2773 [M + Na]^+^ (calcd for C_23_H_42_O_8_Na, 469.2772).

### 3.3. Reagents for Bioassays

Dulbecco′s modified Eagle′s medium (DMEM, 4.5 g/L glucose) was purchased from Sigma-Aldrich (St. Louis, MO, USA). Fetal bovine serum (FBS), penicillin, and streptomycin were purchased from Gibco (Invitrogen, Carlsbad, CA, USA). All other chemicals used in this study were purchased from Wako Pure Chemical Co., Ltd. (Osaka, Japan). The 48- and 96-well microplates (Sumilon) were purchased from Sumitomo Bakelite Co., Ltd. (Tokyo, Japan).

### 3.4. Cell Culture

Murine B16 melanoma 4A5 cells (RCB0557) were obtained from Riken Cell Bank (Tsukuba, Japan). The cells were grown in DMEM supplemented with 10% FBS, penicillin (100 units/mL), and streptomycin (100 µg/mL) at 37 °C in 5% CO_2_/air. The cells were harvested by incubation in phosphate-buffered saline (PBS) containing 0.05% ethylenediaminetetraacetic acid (EDTA) and 0.02% trypsin for ~5 min at 37 °C and used for the subsequent bioassays.

Normal human epidermal melanocytes (NHEMs; Black donor) were obtained from Kurabo (Osaka, Japan), and were cultured in DermaLife Basal Medium (Kurabo, Osaka) supplemented with DermaLife M LifeFactors (Kurabo, Osaka) at 37 °C in 5% CO_2_/air.

### 3.5. Melanogenesis and Cell Viability

The effects on theophylline-stimulated melanogenesis and viability of B16 melanoma 4A5 cells were examined according to previously described protocols [21,22,23,24,25,26,27]. Briefly, murine B16 melanoma 4A5 cells were seeded into 48-well plates in DMEM. After 24 h of culture, a test compound and theophylline (1 mM) were added and incubated for 72 h. The melanin content and cell viability were then measured. IC_50_ values were determined graphically.

Inhibition (%) was calculated using the following formula, where A and B indicate the optical density of the vehicle- and test compound-treated groups, respectively, and C indicates cell viability (%) (see below): Inhibition (%) = [(A − B)/A]/(C/100) × 100.

### 3.6. Melanogenesis in Normal Melanocytes

Effects on melanogenesis in NHEMs were examined according to a protocol described previously [21,22,23,24,25,26,27,34] with modifications. The cells (3.0 × 10^5^ cells/2 mL/well) were seeded into 6-well multiplates. After 24 h of culture, a test compound was added and incubated for 6 days. The cells were harvested using DermaLife Basal Medium supplemented with DermaLife M LifeFactors, and then treated with 1 M NaOH (50 µL/tube, 80 °C, 30 min) to yield a lysate. An aliquot (100 µL) of the lysate was transferred to a 96-well microplate, and the optical density of each well was measured with a microplate reader at 405 nm (reference 655 nm). The test compound was dissolved in DMSO, and the final concentration in the medium was 0.1%. The production rates of melanin were corrected based on the viability of melanoma cells.

### 3.7. AsA Derivative Stability

To determine stability, the samples were stored at 125 °C, and the residual ratio was calculated by measuring the remaining compound by HPLC. HPLC mobile phase conditions were as follows: AsA was described previously, **6** and **20** were 40% methanol containing 0.1% formic acid at flow rate of 0.2 mL/min [52,53].

### 3.8. Mushroom Tyrosinase

Tyrosinase activities using l-tyrosine or l-DOPA as a substrate were determined according to the protocols described previously [20,22,23,24,25,26,27]. Briefly, samples, l-tyrosine (2.5 mM, or l-DOPA, 3.0 mM), mushroom tyrosinase (46 units/mL), and phosphate buffer were mixed in a 96-well plate and incubated at 25 °C. (l-tyrosine; 30 min, l-DOPA; 5 min). The absorbance was then measured at 492 nm.

### 3.9. Mammalian Tyrosinase

Tyrosinase activity using l-DOPA as a substrate was determined according to protocols described previously [50]. Briefly, B16 cells were seeded into 6-well plates in DMEM. After preculture with theophylline (1 mM) for 72 h, the cells were solubilized using 0.1% Triton X-100. Fifty microliters of each lysate and sample was then mixed with 100 µL 2 mM l-DOPA. After the mixtures were incubated for 30 min at 37 °C, the absorbance of each solution at 492 nm was measured.

### 3.10. Expression of Tyrosinase, TRP-1, and TRP-2 mRNA

The expression of tyrosinase, TRP-1, and TRP-2 mRNA was assessed according to previously reported methods [20,23,24,25,26]. Briefly, murine B16 melanoma 4A5 cells were seeded into 96-well plates in DMEM. After 24 h of culture, a test compound and theophylline (1 mM) were added and incubated for 72 h. Then, the amount of each mRNA was quantified by quantitative reverse transcription PCR.

### 3.11. Expression of Tyrosinase Protein

The expression of tyrosinase protein was assessed according to a previously reported method [54]. Briefly, murine B16 melanoma 4A5 cells were seeded into 6-well plates in DMEM. After 24 h of culture, a test compound and theophylline (1 mM) were added and incubated for 72 h. The expression of tyrosinase protein then was measured by western blotting.

### 3.12. Tyrosinase Activity in B16 Cells

Tyrosinase activity in cultured cells was determined according to protocols described previously [34] with modifications. Briefly, B16 melanoma 4A5 cells (4 × 10^3^ cells/100 µL/well) were seeded into 96-well plates in DMEM. After pre-culture with sample and theophylline (1 mM) for 72 h, the cells were solubilized using 0.1% Triton X-100, and then each lysate was mixed with 50 μL 2 mM l-DOPA. After the mixtures were incubated for 1 h at 37 °C, the absorbance of the solution at 492 nm was measured using the microplate reader.

### 3.13. Statistics

Values are expressed as means ± S.D. One-way analysis of variance (ANOVA) followed by Dunnett’s test was used for statistical analysis. Probability (*p*) values less than 0.05 were considered significant.

## 4. Conclusions

**Figure 9:**
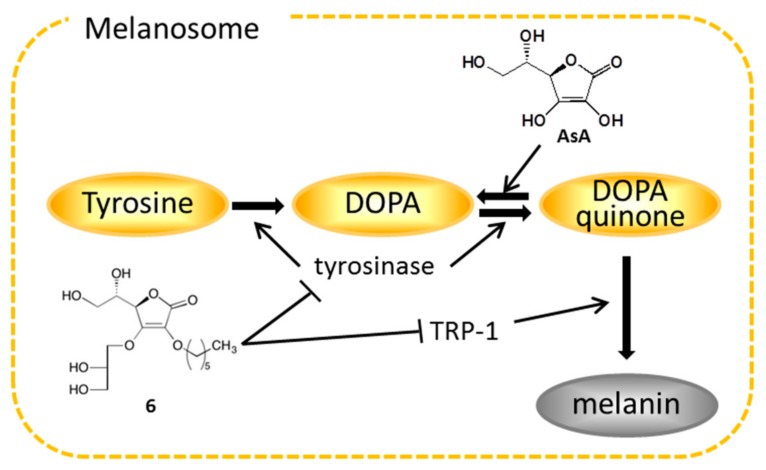
Plausible mechanisms of action of alkylglyceryl-AsA derivatives on melanogenesis inhibitory activity. AsA: l-ascorbic acid.

We examine the inhibitory effects of 28 alkylglyceryl-AsA derivatives (**1**–**28**) on theophylline-stimulated murine B16 melanoma 4A5 cells. We find the following structural requirements to be important for the inhibitory activity of alkylglyceryl-AsA derivatives toward melanogenesis: (i) alkylation of glyceryl-AsA is essential for the activity; (ii) the 3-*O*-alkyl-2-*O*(2,3-dihydroxypropyl)-AsA compounds (**16**–**28**) exhibit stronger activities than those of the corresponding 2-*O*-alkyl-3-*O*-(2,3-dihydroxypropyl)-AsA compounds (**2**–**14**); and (iii) derivatives with longer alkyl chains have significantly stronger inhibitory activity. Using these guidelines along with our cytotoxicity data, 3-*O*-(2,3-dihydroxypropyl)-2-*O*-hexyl-AsA (**6**, IC_50_ = 81.4 µM) and 2-*O*-(2,3-dihydroxypropyl)-3-*O*-hexyl-AsA (**20**, IC_50_ = 117 µM) are deemed the best candidate derivatives for use in cosmetics. However, although it has a limited effective concentration range, 2-*O*-(2,3-dihydroxypropyl)-3-*O*-tetradecyl-AsA (**28**, IC_50_ = 5.0 µM) demonstrates high melanogenesis inhibitory activity. These derivatives are also found to be more stable than AsA and to have favorable characteristics for skin penetration. Mechanistic studies reveal that the mechanisms of action of **6** and **20**, with respect to their melanogenesis inhibitory activities, are the inhibition of tyrosinase and TRP-1 mRNA expression, as well as tyrosinase protein expression, but not the direct inhibition of tyrosinase enzymatic activity (Figure 9). Further skin brightening effects of these candidates, such as those involving intracellular melanosome transport, require further study.

## Figures and Tables

**Table 1:** Inhibitory effects of alkylglyceryl AsA (**1**–**28**) and commercially available AsA derivatives on theophylline-stimulated melanogenesis and viability of B16 4A5 cells.

**Treatment**	**Inhibition (%)**					**IC_50_**
	**0 µM**	**100 µM**	**300 µM**	**1000 µM**	**3000 µM**	**(µM)**
3-*O*-(2,3-Dihydroxypropyl)-AsA (**1**)	0.0 ± 4.1	−11.8 ± 1.9	−10.7 ± 1.1	−5.0 ± 2.9	0.5 ± 2.5	>3000
	(100.0 ± 6.4)	(100.0 ± 1.0)	(96.8 ± 2.3)	(100.4 ± 0.4)	(107.9 ± 1.0)	
3-*O*-(2,3-Dihydroxypropyl)-2-*O*-ethyl-AsA (**2**)	0.0 ± 5.9	−28.1 ± 3.2	−2.8 ± 3.6	−8.9 ± 2.0	40.4 ± 8.6 **	>3000
	(100.0 ± 3.3)	(101.3 ± 3.3)	(99.7 ± 1.8)	(101.3 ± 1.5)	(103.8 ± 4.1)	
3-*O*-(2,3-Dihydroxypropyl)-2-*O*-propyl-AsA (**3**)	0.0 ± 9.2	−2.1 ± 5.3	8.2 ± 3.2	2.5 ± 7.9	35.9 ± 3.4 *	>3000
	(100.0 ± 1.3)	(101.6 ± 1.9)	(98.2 ± 1.7)	(94.5 ± 2.0)	(95.0 ± 1.5)	
2-*O*-Butyl-3-*O*-(2,3-dihydroxy-propyl)-AsA (**4**)	0.0 ± 6.0	−1.2 ± 0.8	20.8 ± 10.6	30.2 ± 5.1 **	58.9 ± 2.7 **	2220
	(100.0 ± 2.1)	(94.5 ± 2.3)	(97.4 ± 0.8)	(93.9 ± 0.8)	(88.7 ± 1.1)	
3-*O*-(2,3-Dihydroxypropyl)-2-*O*-pentyl-AsA (**5**)	0.0 ± 11.4	−14.0 ± 9.7	29.8 ± 8.1	45.9 ± 1.2 **	81.9 ± 3.3 **	931
	(100.0 ± 2.1)	(118.1 ± 2.9)	(107.3 ± 2.3)	(105.1 ± 1.2)	(78.7 ± 1.1 ^#^)	
2-*O*-(2,3-Dihydroxypropyl)-AsA (**15**)	0.0 ± 4.4	−9.7 ± 1.2	−4.8 ± 1.0	−2.3 ± 1.7	−6.1 ± 2.7	>3000
	(100.0 ± 8.5)	(101.2 ± 2.2)	(102.0 ± 3.6)	(101.6 ± 3.2)	(106.7 ± 1.8)	
2-*O*-(2,3-Dihydroxypropyl)-3-*O*-ethyl-AsA (**16**)	0.0 ± 3.0	−4.2 ± 2.3	−5.4 ± 1.5	12.1 ± 2.2 **	42.0 ± 1.4 **	>3000
	(100.0 ± 6.2)	(104.9 ± 1.4)	(95.9 ± 1.2)	(95.9 ± 3.1)	(84.0 ± 1.1)	
2-*O*-(2,3-Dihydroxypropyl)-3-*O*-propyl-AsA (**17**)	0.0 ± 7.4	−5.9 ± 3.3	−3.9 ± 4.1	1.3 ± 4.2	23.2 ± 5.6 **	>3000
	(100.0 ± 4.3)	(98.4 ± 4.7)	(95.6 ± 3.5)	(92.0 ± 2.3)	(82.9 ± 2.3)	
3-*O*-Butyl-2-*O*-(2,3-dihydroxy-propyl)-AsA (**18**)	0.0 ± 2.7	0.2 ± 4.4	36.3 ± 2.8 **	68.0 ± 2.1 **	84.1 ± 1.4 **	473
	(100.0 ± 1.0)	(99.8 ± 1.1)	(89.1 ± 1.6)	(78.1 ± 0.5 ^#^)	(70.6 ± 1.0 ^#^)	
2-*O*-(2,3-Dihydroxypropyl)-3-*O*-pentyl-AsA (**19**)	0.0 ± 5.2	14.0 ± 4.4 *	53.0 ± 1.1 **	83.1 ± 1.4 **	97.1 ± 0.9 **	283
	(100.0 ± 0.4)	(86.7 ± 1.5)	(73.4 ± 0.7 ^#^)	(54.7 ± 0.8 ^#^)	(22.5 ± 0.5 ^#^)	
2-*O*-(2,3-Dihydroxypropyl)-3-*O*-hexyl-AsA (**20**)	0.0 ± 2.9	43.5 ± 2.6 **	77.0 ± 2.1 **	94.9 ± 1.5 **	80.9 ± 4.1 **	117
	(100.0 ± 1.0)	(95.3 ± 0.6)	(83.9 ± 0.4)	(60.8 ± 0.6 ^#^)	(44.7 ± 1.9 ^#^)	
AsA	0.0 ± 1.7	—	—	−17.4 ± 4.0	3.8 ± 4.7	>3000
	(100.0 ± 2.2)			(103.8 ± 0.8)	(89.2 ± 0.8)	
Magnesium l-ascorbyl-2-phosphate	0.0 ± 5.5	—	—	2.1 ± 4.1	14.5 ± 1.3 *	>3000
	(100.0 ± 0.5)			(125.6 ± 3.6)	(92.8 ± 3.0)	
2-*O*-α-d-Glucopyranosyl-AsA	0.0 ± 3.0	—	—	−8.9 ± 2.7	15.0 ± 3.5 *	>3000
	(100.0 ± 4.3)			(106.8 ± 2.2)	(108.4 ± 5.4)	
Erythrobic acid	0.0 ± 6.7	−22.9 ± 6.5	−10.6 ± 4.8	75.8 ± 4.2 **	92.6 ± 15.3 **	—
	(100.0 ± 5.7)	(98.7 ± 4.3)	(94.8 ± 3.6)	(44.5 ± 2.0 ^#^)	(29.8 ± 2.2 ^#^)	
Arbutin	0.0 ± 10.0	32.2 ± 3.4 **	22.3 ± 4.3 **	63.0 ± 2.3 **	94.0 ± 2.8 **	830
	(100.0 ± 3.7)	(92.2 ± 0.6)	(96.0 ± 2.0)	(96.2 ± 2.7)	(105.8 ± 2.9)	
**Treatment**	**Inhibition (%)**					**IC_50_**
	**0 µM**	**3 µM**	**10 µM**	**30 µM**	**100 µM**	**(µM)**
3-*O*-(2,3-Dihydroxypropyl)-2-*O*-hexyl-AsA (**6**)	0.0 ± 5.7	−1.8 ± 4.9	5.6 ± 2.0	26.2 ± 6.0 **	53.1 ± 3.1 **	81.4
	(100.0 ± 6.0)	(102.1 ± 4.9 ^#^)	(96.1 ± 4.9)	(88.4 ± 6.6)	(77.1 ± 5.8 ^#^)	
3-*O*-(2,3-Dihydroxypropyl)-2-*O*-heptyl-AsA (**7**)	0.0 ± 9.5	−4.2 ± 6.3	3.4 ± 5.2	20.0 ± 6.4 *	52.2 ± 5.0 **	89.1
	(100.0 ± 0.7)	(100.6 ± 3.0)	(95.4 ± 3.6)	(91.9 ± 4.7)	(79.7 ± 2.9 ^#^)	
3-*O*-(2,3-Dihydroxypropyl)-2-*O*-octyl-AsA (**8**)	0.0 ± 8.3	5.7 ± 6.9	10.6 ± 4.6 *	22.4 ± 4.4 **	64.1 ± 8.2 **	68.8
	(100.0 ± 5.1)	(108.4 ± 6.2)	(102.8 ± 4.8)	(92.5 ± 4.6)	(80.0 ± 4.4)	
3-*O*-(2,3-Dihydroxypropyl)-2-*O*-nonyl-AsA (**9**)	0.0 ± 5.9	0.2 ± 4.4	19.1 ± 1.3 **	51.9 ± 4.6 **	91.6 ± 1.2 **	28.8
	(100.0 ± 2.3)	(99.2 ± 0.5)	(88.9 ± 4.2)	(77.6 ± 3.6 ^#^)	(58.4 ± 2.3 ^#^)	
2-*O*-Decyl-3-*O*-(2,3-dihydroxy-propyl)-AsA (**10**)	0.0 ± 6.7	3.7 ± 7.8	39.2 ± 4.1 **	78.1 ± 4.8 **	98.0 ± 3.7 **	13.0
	(100.0 ± 4.3)	(98.6 ± 4.3)	(85.9 ± 2.3)	(73.5 ± 5.8 ^#^)	(27.9 ± 2.8 ^#^)	
3-*O*-(2,3-Dihydroxypropyl)-2-*O*-undecyl-AsA (**11**)	0.0 ± 2.9	12.4 ± 3.0 **	35.4 ± 2.8 **	89.3 ± 1.5 **	100.3 ± 4.7 **	15.1
	(100.0 ± 4.8)	(106.2 ± 2.5)	(90.8 ± 7.1)	(69.0 ± 1.9 ^#^)	(25.8 ± 1.9 ^#^)	
3-*O*-(2,3-Dihydroxypropyl)-2-*O*-dodecyl-AsA (**12**)	0.0 ± 5.3	20.4 ± 10.2 *	50.0 ± 5.7 **	96.0 ± 2.9 **	97.9 ± 4.2 **	10.6
	(100.0 ± 7.3)	(112.1 ± 5.8)	(103.8 ± 1.2)	(73.3 ± 2.1 ^#^)	(24.6 ± 3.4 ^#^)	
3-*O*-(2,3-Dihydroxypropyl)-2-*O*-tridecyl-AsA (**13**)	0.0 ± 5.2	0.1 ± 8.6	46.0 ± 7.9 **	97.1 ± 2.3 **	107.8 ± 9.1 **	11.3
	(100.0 ± 2.7)	(96.3 ± 3.5)	(87.2 ± 1.8)	(63.7 ± 1.1 ^#^)	(23.5 ± 2.1 ^#^)	
3-*O*-(2,3-Dihydroxypropyl)-2-*O*-tetradecyl-AsA (**14**)	0.0 ± 7.1	6.3 ± 2.6	48.4 ± 2.2 **	97.6 ± 1.8 **	100.0 ± 18.2 **	11.1
	(100.0 ± 2.3)	(101.2 ± 1.8)	(89.1 ± 4.5)	(56.3 ± 3.2 ^#^)	(21.0 ± 2.4 ^#^)	
2-*O*-(2,3-Dihydroxypropyl)-3-*O*-heptyl-AsA (**21**)	0.0 ± 2.8	−6.3 ± 2.7 *	0.9 ± 8.2	21.0 ± 5.0 **	44.6 ± 6.2 **	>100
	(100.0 ± 7.1)	(97.9 ± 0.9)	(92.7 ± 3.7)	(92.7 ± 4.2)	(86.3 ± 2.7)	
2-*O*-(2,3-Dihydroxypropyl)-3-*O*-octyl-AsA (**22**)	0.0 ± 8.8	−10.4 ± 6.0	1.0 ± 7.0	2.3 ± 5.9	34.8 ± 8.2 **	>100
	(100.0 ± 2.0)	(98.8 ± 3.4)	(99.3 ± 5.2)	(91.6 ± 4.0)	(83.0 ± 4.9)	
2-*O*-(2,3-Dihydroxypropyl)-3-*O*-nonyl-AsA (**23**)	0.0 ± 14.8	−1.3 ± 9.4	−2.6 ± 8.0	14.9 ± 5.6	77.1 ± 2.4 **	72.9
	(100.0 ± 2.4)	(96.6 ± 5.5)	(90.6 ± 2.2)	(81.2 ± 4.7)	(60.7 ± 3.2 ^#^)	
3-*O*-Decyl-2-*O*-(2,3-dihydroxy-propyl)-AsA (**24**)	0.0 ± 5.0	−5.8 ± 6.0	20.9 ± 5.4 **	64.3 ± 3.8 **	102.7 ± 2.4 **	23.5
	(100.0 ± 3.2)	(92.5 ± 5.7)	(87.1 ± 1.6)	(68.5 ± 2.6 ^#^)	(34.3 ± 1.5 ^#^)	
2-*O*-(2,3-Dihydroxypropyl)-3-*O*-undecyl-AsA (**25**)	0.0 ± 2.0	−5.1 ± 2.3 *	26.6 ± 3.0 **	85.0 ± 3.8 **	100.8 ± 10.1 **	18.1
	(100.0 ± 4.8)	(101.0 ± 2.9)	(85.8 ± 3.5)	(53.6 ± 2.7 ^#^)	(24.0 ± 0.5 ^#^)	
2-*O*-(2,3-Dihydroxypropyl)-3-*O*-dodecyl-AsA (**26**)	0.0 ± 7.9	14.1 ± 8.5 *	46.0 ± 3.0 **	96.2 ± 2.8 **	89.6 ± 24.4 **	12.1
	(100.0 ± 7.2)	(99.8 ± 4.2)	(87.3 ± 2.8)	(45.5 ± 1.1 ^#^)	(24.3 ± 12.7 ^#^)	
2-*O*-(2,3-Dihydroxypropyl)-3-*O*-tridecyl-AsA (**27**)	0.0 ± 8.4	20.1 ± 5.7 **	44.2 ± 3.9 **	96.6 ± 3.0 **	112.9 ± 10.1 **	11.7
	(100.0 ± .5.5)	(104.9 ± 4.1)	(84.3 ± 2.8)	(40.2 ± 2.5 ^#^)	(21.0 ± 1.5 ^#^)	
2-*O*-(2,3-Dihydroxypropyl)-3-*O*-tetradecyl-AsA (**28**)	0.0 ± 7.6	32.8 ± 7.2 **	75.7 ± 3.9 **	95.9 ± 8.4 **	95.2 ± 20.7 **	5.0
	(100.0 ± 1.3)	(85.1 ± 4.6)	(62.6 ± 1.6 ^#^)	(23.0 ± 2.1 ^#^)	(18.1 ± 1.9 ^#^)	
Hydroquinone	0.0 ± 4.4	37.4 ± 3.7 **	59.5 ± 3.7 **	76.3 ± 2.1 **	—	8.7
	(100.0 ± 1.6)	(94.1 ± 1.7)	(85.2 ± 1.4)	(64.3 ± 0.8 ^#^)		

Each value represents the mean ± S.D. (*n* = 4); asterisks denote significant differences from the control group, * *p* < 0.05, ** *p* < 0.01; ^#^ cytotoxic effects were observed, and values in parentheses indicate cell viability (%). —: not measured; AsA: l-ascorbic acid.

**Table 2:** Effects on activity of tyrosinase from mushroom.

**Treatment**	**Inhibition (%)**					
	**Substrate: l-Tyrosine**			**Substrate: l-DOPA**		
	**0 µM**	**30 µM**	**100 µM**	**0 µM**	**30 µM**	**100 µM**
3-*O*-(2,3-Dihydroxypropyl)-2-*O*-hexyl-AsA (**6**)	0.0 ± 0.4	−1.5 ± 6.7	0.9 ± 1.4	0.0 ± 6.3	−1.5 ± 1.5	−1.0 ± 0.7
3-*O*-(2,3-Dihydroxypropyl)-2-*O*-heptyl-AsA (**7**)	0.0 ± 0.4	−1.7 ± 4.0	1.0 ± 2.4	0.0 ± 6.3	−0.7 ± 3.1	1.1 ± 4.8
3-*O*-(2,3-Dihydroxypropyl)-2-*O*-octyl-AsA (**8**)	0.0 ± 0.4	−0.3 ± 2.3	−0.6 ± 4.3	0.0 ± 2.4	2.4 ± 1.1	2.3 ± 2.7
3-*O*-(2,3-Dihydroxypropyl)-2-*O*-nonyl-AsA (**9**)	0.0 ± 0.4	4.0 ± 6.1	−1.2 ± 4.5	0.0 ± 2.4	4.8 ± 6.6	0.9 ± 6.0
2-*O*-(2,3-Dihydroxypropyl)-3-*O*-hexyl-AsA (**20**)	0.0 ± 10.7	1.4 ± 4.7	−6.1 ± 0.6	0.0 ± 8.7	−5.7 ± 5.1	−2.4 ± 4.2
2-*O*-(2,3-Dihydroxypropyl)-3-*O*-heptyl-AsA (**21**)	0.0 ± 10.7	−9.2 ± 2.1	−9.8 ± 3.9	0.0 ± 8.7	−10.0 ± 1.7	−11.3 ± 2.4
2-*O*-(2,3-Dihydroxypropyl)-3-*O*-octyl-AsA (**22**)	0.0 ± 10.7	−11.5 ± 1.6	−14.4 ± 1.8	0.0 ± 11.2	−13.1 ± 3.3	−4.5 ± 2.0
2-*O*-(2,3-Dihydroxypropyl)-3-*O*-nonyl-AsA (**23**)	0.0 ± 7.0	−5.0 ± 3.1	−2.6 ± 2.4	0.0 ± 2.2	−4.1 ± 2.2	−1.5 ± 2.2
	**0 µM**	**10 µM**	**30 µM**	**0 µM**	**10 µM**	**30 µM**
2-*O*-Decyl-3-*O*-(2,3-dihydroxy-propyl)-AsA (**10**)	0.0 ± 0.4	1.3 ± 3.1	0.4 ± 1.7	0.0 ± 2.4	0.4 ± 1.1	2.4 ± 2.9
3-*O*-(2,3-Dihydroxypropyl)-2-*O*-undecyl-AsA (**11**)	0.0 ± 0.4	2.9 ± 3.9	1.0 ± 2.5	0.0 ± 3.2	−4.6 ± 1.4	−5.1 ± 2.2
3-*O*-(2,3-Dihydroxypropyl)-2-*O*-dodecyl-AsA (**12**)	0.0 ± 6.7	−0.7 ± 4.5	−7.1 ± 1.4	0.0 ± 3.2	−3.1 ± 3.3	−4.6 ± 2.2
3-*O*-(2,3-Dihydroxypropyl)-2-*O*-tridecyl-AsA (**13**)	0.0 ± 6.7	−4.7 ± 2.9	−7.1 ± 1.7	0.0 ± 3.2	−4.7 ± 5.0	−4.4 ± 1.8
3-*O*-(2,3-Dihydroxypropyl)-2-*O*-tetradecyl-AsA (**14**)	0.0 ± 6.7	−7.0 ± 6.7	−9.7 ± 2.7	0.0 ± 13.8	−8.9 ± 3.5	−8.2 ± 4.3
3-*O*-Decyl-2-*O*-(2,3-dihydroxy-propyl)-AsA (**24**)	0.0 ± 7.0	−4.1 ± 5.3	−3.1 ± 4.1	0.0 ± 4.2	0.7 ± 3.8	−5.3 ± 4.2
2-*O*-(2,3-Dihydroxypropyl)-3-*O*-undecyl-AsA (**25**)	0.0 ± 7.0	−8.0 ± 0.6	−8.7 ± 1.5	0.0 ± 4.2	−10.2 ± 2.3	−10.7 ± 3.8
2-*O*-(2,3-Dihydroxypropyl)-3-*O*-dodecyl-AsA (**26**)	0.0 ± 7.0	−2.2 ± 6.4	−0.5 ± 4.3	0.0 ± 3.0	−6.4 ± 3.1	−6.6 ± 8.7
2-*O*-(2,3-Dihydroxypropyl)-3-*O*-tridecyl-AsA (**27**)	0.0 ± 7.0	−3.3 ± 8.1	−5.4 ± 1.9	0.0 ± 3.0	−2.0 ± 3.3	−3.2 ± 2.7
2-*O*-(2,3-Dihydroxypropyl)-3-*O*-tetradecyl-AsA (**28**)	0.0 ± 7.0	−4.4 ± 4.6	−8.4 ± 1.0	0.0 ± 3.0	−5.7 ± 9.5	−8.1 ± 5.2
**Substrate: l-Tyrosine**	**Inhibition (%)**					
**Treatment**	**0 µM**	**10 µM**	**30 µM**	**100 µM**	**300 µM**	**IC_50_ (µM)**
Kojic acid [20,22,23,24,25,26,27]	0.0 ± 2.4	12.2 ± 3.3	46.4 ± 2.6 **	66.5 ± 2.1 **	96.8 ± 0.9 **	43.6
**Substrate: l-DOPA**	**Inhibition (%)**					
**Treatment**	**0 µM**	**10 µM**	**30 µM**	**100 µM**	**300 µM**	**IC_50_ (µM)**
Kojic acid [20,22,23,24,25,26,27]	0.0 ± 0.9	22.3 ± 2.1 **	50.6 ± 0.6 **	78.2 ± 0.7 **	89.3 ± 0.3 **	29.6

Each value represents the mean ± S.D. (*n* = 4); asterisks denote significant differences from the control group, ** *p* < 0.01. AsA: l-ascorbic acid.

**Table 3:** Effects of **6**, **14**, **20**, and **28** on expression of tyrosinase, TRP-1, and TRP-2 mRNA in B16 4A5 cells.

**Treatment**	**Tyrosinase mRNA/β-actin mRNA**		
	**0 µM**	**30 µM**	**100 µM**
3-*O*-(2,3-Dihydroxypropyl)-2-*O*-hexyl-AsA (**6**)	1.00 ± 0.05	0.60 ± 0.07 **	0.42 ± 0.03 **
2-*O*-(2,3-Dihydroxypropyl)-3-*O*-hexyl-AsA (**20**)	1.00 ± 0.22	0.72 ± 0.10	0.59 ± 0.07 *
**Treatment**	**TRP-1 mRNA/β-actin mRNA**		
	**0 µM**	**30 µM**	**100 µM**
3-*O*-(2,3-Dihydroxypropyl)-2-*O*-hexyl-AsA (**6**)	1.00 ± 0.21	0.48 ± 0.15 *	0.37 ± 0.05 **
2-*O*-(2,3-Dihydroxypropyl)-3-*O*-hexyl-AsA (**20**)	1.00 ± 0.21	0.67 ± 0.12	0.50 ± 0.15 *
**Treatment**	**TRP-2 mRNA/β-actin mRNA**		
	**0 µM**	**30 µM**	**100 µM**
3-*O*-(2,3-Dihydroxypropyl)-2-*O*-hexyl-AsA (**6**)	1.00 ± 0.32	0.53 ± 0.18	0.70 ± 0.06
2-*O*-(2,3-Dihydroxypropyl)-3-*O*-hexyl-AsA (**20**)	1.00 ± 0.10	1.07 ± 0.30	0.88 ± 0.25
**Treatment**	**Tyrosinase mRNA/*β*-actin mRNA**		
	**0 µM**	**3 µM**	**10 µM**
3-*O*-(2,3-Dihydroxypropyl)-2-*O*-tetradecyl-l-ascorbic acid (**14**)	1.00 ± 0.12	0.78 ± 0.10	0.58 ± 0.09 **
2-*O*-(2,3-Dihydroxypropyl)-3-*O*-tetradecyl-l-ascorbic acid (**28**)	1.00 ± 0.12	0.54 ± 0.03 **	0.29 ± 0.08 **
**Treatment**	**TRP-1 mRNA/*β*-actin mRNA**		
	**0 µM**	**3 µM**	**10 µM**
3-*O*-(2,3-Dihydroxypropyl)-2-*O*-tetradecyl-l-ascorbic acid (**14**)	1.00 ± 0.24	1.02 ± 0.27	0.92 ± 0.22
2-*O*-(2,3-Dihydroxypropyl)-3-*O*-tetradecyl-l-ascorbic acid (**28**)	1.00 ± 0.24	0.88 ± 0.20	0.83 ± 0.27
**Treatment**	**TRP-2 mRNA/*β*-actin mRNA**		
	**0 µM**	**3 µM**	**10 µM**
3-*O*-(2,3-Dihydroxypropyl)-2-*O*-tetradecyl-l-ascorbic acid (**14**)	1.00 ± 0.11	0.58 ± 0.06 **	0.50 ± 0.08 **
2-*O*-(2,3-Dihydroxypropyl)-3-*O*-tetradecyl-l-ascorbic acid (**28**)	1.00 ± 0.11	0.43 ± 0.11 **	0.35 ± 0.05 **

Each value represents the mean ± S.D. (*n* = 3); asterisks denote significant differences from the control group, * *p* < 0.05, ** *p* < 0.01. AsA: l-ascorbic acid.

## Supplementary Materials

Supplementary Materials are available at http://www.mdpi.com/1422-0067/19/4/1144/s1.
