# Supplementary material for: Structural Requirements of Alkylglyceryl-l-Ascorbic Acid Derivatives for Melanogenesis Inhibitory Activity
Source: Int J Mol Sci. 2018 Apr 10;19(4):1144. doi: 10.3390/ijms19041144 (PMC5979531; doi:10.3390/ijms19041144)
Supplement: Supplementary file 1 [file ijms-19-01144-s001.pdf]

Supplementary Materials: Structural Requirements of Alkylglyceryl-L-Ascorbic Acid Derivatives for Melanogenesis Inhibitory Activity

Norihisa Taira *, Yushi Katsuyama , Masato Yoshioka , Osamu Muraoka , and Toshio Morikawa

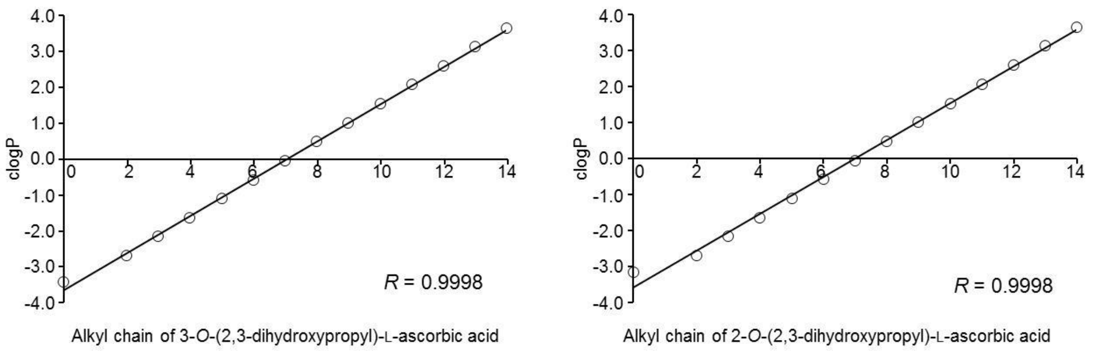

Figure S1. Correlations between *c*log P values and length of the alkyl chain in 2-*O*-alkyl-3-*O*-(2,3-dihydroxypropyl)-L-ascorbic acids (**2**–**14**) and 3-*O*-alkyl-2-*O*-(2,3-dihydroxypropyl)-L-ascorbic acids (**16**–**28**). *c*log P Values were calculated by ChemDraw Ultra ver. 7.0.1 software (PerkinElmer Inc., Waltham, MA, USA).
